# Three-Dimensional Boundary Element Strategy for Stress Sensitivity of Fractional-Order Thermo-Elastoplastic Ultrasonic Wave Propagation Problems of Anisotropic Fiber-Reinforced Polymer Composite Material

**DOI:** 10.3390/polym14142883

**Published:** 2022-07-16

**Authors:** Mohamed Abdelsabour Fahmy

**Affiliations:** 1 Adham University College, Umm Al-Qura University, Makkah 28653, Saudi Arabia; maselim@uqu.edu.sa; Tel.: +966-537-930-306; 2 Faculty of Computers and Informatics, Suez Canal University, New Campus, Ismailia 41522, Egypt; mohamed_fahmy@ci.suez.edu.eg

**Keywords:** boundary element method, stress sensitivity, fractional-order thermo-elastoplastic, ultrasonic wave propagation problems, fiber-reinforced polymer composite materials

## Abstract

A new three-dimensional (3D) boundary element method (BEM) strategy was developed to solve fractional-order thermo-elastoplastic ultrasonic wave propagation problems based on the meshless moving least squares (MLS) method. The temperature problem domain was divided into a number of circular sub-domains. Each node was the center of the circular sub-domain surrounding it. The Laplace transform method was used to solve the temperature problem. A unit test function was used in the local weak-form formulation to generate the local boundary integral equations, and the inverse Laplace transformation method was used to find the transient temperature solutions. Then, the three-dimensional elastoplastic problems could be solved using the boundary element method (BEM). Initial stress and strain formulations are adopted, and their distributions are interpolated using boundary integral equations. The effects of the fractional-order parameter and anisotropy are investigated. The proposed method’s validity and performance are demonstrated for a two-dimensional problem with excellent agreement with other experimental and numerical results.

## 1. Introduction

All fiber-reinforced polymer (FRP) composite materials, which have significant potential for a wide range of infrastructure applications, contain thermosetting or thermoplastic resins as well as glass and/or carbon fibers. The load-bearing component of the composite is provided by the fiber network, while the resin aids in load transfer and fiber orientation. The resin regulates the manufacturing process and processing variables. Resins also protect the fabrics from environmental factors such as relative humidity-elevated temperatures and chemical attacks.

Significant research has been conducted on the development of FRP composite materials and their novel applications. Many efforts have yielded materials with improved structural properties. Because of their superior corrosion resistance, excellent thermomechanical properties, and high strength-to-weight ratio, FRP composite materials are being promoted as twenty-first-century materials. In terms of their embodied energy, FRP composite materials are also “greener” than traditional materials such as concrete and steel. The use of FRP composite materials in civil and military infrastructure can improve innovation, productivity, and performance while also providing longer service lives, resulting in lower life-cycle costs. These efforts demonstrate that the use of innovative composite materials and designs have significant potential to reduce infrastructure vulnerability.

The BEM with internal collocation nodes has been used to solve thermo-elastoplastic problems [1,2]. However, the BEM’s advantage of ease of data preparation is lost in this scenario. Therefore, several BEM strategies have been proposed. Nowak and Neves [3] developed the multiple-reciprocity boundary element method, which cannot be used to analyze thermo-elastoplastic materials. The dual-reciprocity BEM was developed to solve thermo-elastoplastic problems with an arbitrary heat source [4]. Eigenvalue analysis can be carried out using the real-part boundary element approach [5,6]. The local boundary element method was used by Sladek and Sladek [7] to solve elastoplastic problems without internal cells. For elastoplastic difficulties, Ochiai and Kobayashi [8] presented the triple-reciprocity BEM, which does not require internal cells. This method allows for a very accurate solution to be produced using only fundamental low-order solutions and reduces the requirements for data preparation. Ochiai [9] applied the triple-reciprocity BEM to solve 2D thermo-elastoplastic problems with an arbitrary distributed heat source [10] and three-dimensional elastoplastic problems with initial strain formulas [10]. Recently, Fahmy et al. [11,12,13,14] developed fractional BEM schemes to solve certain thermoelastic problems.

In this paper, a new BEM strategy is developed to solve three-dimensional thermo-elastoplastic wave propagation problems with an arbitrary distributed heat source. Boundary elements and arbitrary internal points are used in this strategy. For elastoplastic analysis, the initial strain or stress distribution is interpolated using boundary integral equations. Strong singularities in the calculation of stresses at internal sites become weak using this method. The impacts of anisotropy and the fractional-order parameter are examined. The validity and performance of the suggested method for a two-dimensional problem are demonstrated, showing excellent agreement with existing experimental and numerical results.

## 2. BEM Implementation for the Temperature Field

The heat conduction equation of a nonhomogeneous anisotropic fiber-reinforced polymer composite in the presence of the distributed heat source $W^{\left[1\right]s}\left(q\right)$ can be expressed as [15]

(1) $$\rho \left(x\right)c\left(x\right)D_{t}^{\tilde{\alpha }}\theta \left(x,t\right)={\left[k_{ij}\left(x\right)\theta_{,j}\left(x,t\right)\right]}_{,i}+Q\left(x,t\right),\;$$

in which

$$Q\left(x,t\right)=\frac{1-R}{x_{0}}W^{\left[1\right]s}\left(q\right)\;e^{\left(-\frac{x_{a}}{x_{0}}\right)J\left(t\right)},\;J\left(t\right)=\frac{J_{0}\;t}{\tau_{1}^{2}}e^{-\frac{t}{\tau_{1}}},\;a=1,\;2,\;3$$

where the parameters are defined in the Nomenclature Table at the end of this paper.

In the BEM formulation of 3D problems, the distributed heat source function $W_{1}^{S}\left(q\right)$ is interpolated using the following equations [16]:

(2) $$\nabla^{2}W^{\left[1\right]S}\left(q\right)=-W^{\left[2\right]S}\left(q\right),$$

(3) $$\nabla^{2}W^{\left[2\right]S}\left(q\right)=-\overset{M}{\underset{m=1}{\sum }}W^{\left[3\right]PA}\left(q_{m}\right),\;$$

In 3D problems, the polyharmonic function with the volume distribution $T^{\left[f\right]A}\left(p,q\right)$ is introduced to achieve smooth interpolation and can be described as [17]

(4) $$\begin{array}{l} T^{[f|A}\left(p,q\right)=\frac{1}{2r\left(2f+1\right)!}\left\{\left(2fA-r\right){\left(r+A\right)}^{2f}+\left(2fA+r\right){\left(r-A\right)}^{2f}\right\}\;\;r>A,\; \end{array}\;$$

(5) $$\begin{array}{c} T^{[f|A}\left(p,q\right)=\frac{1}{2r\left(2f+1\right)!}\left\{\left(2fA-r\right){\left(A+r\right)}^{2f}-\left(2fA+r\right){\left(A-r\right)}^{2f}\right\}\;\;r\leq A.\; \end{array}$$

where r denotes the distance between observation point p and loading point q.

On the basis of Caputo’s finite difference technique, at $\left(f+1\right)\Delta \tau$ and fΔτ, the following formula can be established [18]:

(6) $$D_{\tau }^{\tilde{\alpha }}\theta^{f+1}+D_{\tau }^{\tilde{\alpha }}\theta^{f}\approx \overset{k}{\underset{J=0}{\sum }}W_{\tilde{\alpha },J}\left(\theta^{f+1-J}\left(x\right)-\theta^{f-J}\left(x\right)\right)\;$$

where

(7) $$W_{\tilde{\alpha },0}=\frac{{\left(\Delta \tau \right)}^{-\tilde{\alpha }}}{\Gamma \left(2-\tilde{\alpha }\right)}\;and\;W_{\tilde{\alpha },J}=W_{\tilde{\alpha },0}\left({\left(J+1\right)}^{1-\tilde{\alpha }}-{\left(J-1\right)}^{1-\tilde{\alpha }}\right)\;$$

By employing Equation (6), the fractional nonlinear heat conduction Equation (1) is transformed into the following equation [19]:

(8) $$W_{\tilde{\alpha },0}\theta^{f+1}\left(x\right)-\lambda \left(x\right)\theta_{,ii}^{f+1}\left(x\right)-\lambda_{,i}\left(x\right)\theta_{,i}^{f+1}\left(x\right)=W_{\tilde{\alpha },0}\theta^{f}\left(x\right)-\lambda \left(x\right)\theta_{,ii}^{f}\;\left(x\right)\;-\lambda_{,i}\left(x\right)\theta_{,j}^{f}\;\left(x\right)-\overset{f}{\underset{J=1}{\sum }}W_{\tilde{\alpha },J}\left(\theta^{f+1-J}\left(x\right)-\theta^{f-J}\left(x\right)\right)+h_{m}^{f+1}\left(x,t\right)+h_{m}^{f}\left(x,t\right)\;$$

Let Ω be the analyzed domain of the considered problem and the initial condition be

(9) $${\left.\theta \left(x,t\right)\right|}_{t=0}=\theta \left(x,0\right)$$

The MLS approximates $u^{h}\left(x\right)$ as $u^{h}\left(x\right)=p^{T}\left(x\right)a\left(x\right)\;\;\forall x\in \Omega_{x}$, where $p^{T}\left(x\right)=\left[p^{1}\left(x\right),p^{2}\left(x\right),\ldots ,p^{m}\left(x\right)\right]$, and $a\left(x\right)$ is a vector of coefficients $a^{j}\left(x\right),\;\left(j=1,2,\ldots ,m\right)$, $x={\left[x_{1},x_{2},x_{3}\right]}^{T}$. Thus, the following definitions can be deduced:

(10) $$\begin{array}{ll} p^{T}\left(x\right)= & \left[1,x_{1},x_{2},x_{3}\right],\;\mathrm{linear}\;\mathrm{basis}\;m=4,\\ p^{T}\left(x\right)= & \left[1,x_{1},x_{2},x_{3},x_{1}^{2},x_{2}^{2},x_{3}^{2},x_{1}x_{2},x_{2}x_{3},x_{3}x_{1}\right],\;\mathrm{quatratic}\;\mathrm{basis}\;m=10. \end{array}$$

Now, by implementing the Laplace transformation to Equation (1), the following equation is obtained:

(11) $${\left[k_{ij}\left(x\right){\bar{\theta }}_{,j}\left(x,s\right)\right]}_{,\;i}-\rho \left(x\right)c\left(x\right)s\bar{\theta }\left(x,s\right)=-\bar{F}\left(x,s\right),\;$$

in which

(12) $$\bar{F}\left(x,s\right)=\bar{Q}\left(x,s\right)+\theta \left(x,0\right)\;$$

where $\bar{Q}\left(x,s\right)=\frac{1-R}{x_{0}}e^{\frac{x_{a}}{x_{0}}J\left(s\right)}$, and $J\left(s\right)=\frac{J_{0}}{{\left(s+\tau_{1}\right)}^{2}},\;s>\tau_{1}$.

The local weak form of Equation (11) can be described as

(13) $$\int_{\Omega_{s}^{a}}\left[{\left(k_{lj}\left(x\right){\bar{\theta }}_{,j}\left(x,s\right)\right)}_{,l}-\rho \left(x\right)c\left(x\right)s\bar{\theta }\left(x,s\right)+\bar{F}\left(x,s\right)\right]\theta^{*}\left(x\right)d\Omega =0,\;x^{a}\in \Omega_{s}^{a}$$

in which $\theta^{*}\left(x\right)$ and $\partial \Omega_{s}^{a}$ are the weight function and local sub-domain boundary, respectively.

Applying the Gauss theorem to Equation (13) yields

(14) $$\begin{array}{c} \int_{\partial \Omega_{s}^{a}}\bar{q}\left(x,s\right)\theta^{*}\left(x\right)d\Gamma -\int_{\Omega_{s}^{a}}k_{lj}\left(x\right){\bar{\theta }}_{,j}\left(x,s\right)\theta_{,l}^{*}\left(x\right)d\Omega \\ -\int_{\Omega_{s}^{a}}\rho \left(x\right)c\left(x\right)s\bar{\theta }\left(x,s\right)\theta^{*}\left(x\right)d\Omega +\int_{\Omega_{s}^{a}}\bar{F}\left(x,s\right)\theta^{*}\left(x\right)d\Omega =0, \end{array}$$

where

(15) $$\bar{q}\left(x,s\right)=k_{lj}\left(x\right){\bar{\theta }}_{,j}\left(x,s\right)n_{l}\left(x\right).\;$$

and

(16) $$\theta^{*}\left(x\right)=\left\{\begin{array}{ll} 1 & \;\mathrm{at}\;x\in \Omega_{s}^{a}\\ 0 & \;\mathrm{at}\;x\notin \Omega_{s}^{a} \end{array}\right.\;$$

Based on the fundamental solution of (8), the local weak form (14) yields the following boundary integral representation:

(17) $$\int_{\partial \Omega_{s}^{a}}\bar{q}\left(x,s\right)d\Gamma -\int_{\Omega_{s}^{a}}\rho \left(x\right)c\left(x\right)s\bar{\theta }\left(x,s\right)d\Omega =-\int_{\Omega_{s}^{a}}\bar{F}\left(x,s\right)d\Omega .$$

The MLS is employed to compute the heat flux as

(18) $${\bar{q}}^{h}\left(x,s\right)=k_{ij}n_{i}\overset{n}{\underset{a=1}{\sum }}\phi_{,j}^{a}\left(x\right){\hat{\theta }}^{a}\left(s\right).$$

On the basis of [20], Equation (17) can be re-expressed as

(19) $$\begin{array}{ll} \overset{n}{\underset{a=1}{\sum }} & \left(\int_{L_{s}+\Gamma_{sp}}n^{T}KP^{a}\left(x\right)d\Gamma -\int_{\Omega_{s}}\rho \mathrm{cs}\phi^{a}\left(x\right)d\Gamma \right){\hat{\theta }}^{a}\left(s\right)\\ & =-\int_{\Gamma_{sq}}\tilde{\bar{q}}\left(x,s\right)d\Gamma -\int_{\Omega_{s}}\bar{R}\left(x,s\right)d\Omega , \end{array}$$

Considering the following representations

(20) $$K=\left[\begin{array}{lll} k_{11} & k_{12} & k_{13}\\ k_{12} & k_{22} & k_{23}\\ k_{13} & k_{23} & k_{33} \end{array}\right],\;P^{a}\left(x\right)=\left[\begin{array}{c} \phi_{,1}^{a}\\ \phi_{,2}^{a}\\ \phi_{,3}^{a} \end{array}\right],\;n^{T}=\left(n_{1},n_{2},n_{3}\right).\;$$

The inverse Laplace transform [21] has now been implemented to obtain the physical quantities in time domain.

## 3. BEM Implementation for the Elastoplastic Field

Now, our purpose is to solve the following boundary integral equation [1,2]:

(21) $$\begin{array}{ll} c_{ij}\left(P\right){\dot{u}}_{j}\left(P\right)= & \underset{\Gamma }{\int }\left[u_{ij}^{\left[1\right]}\left(P,Q\right){\dot{p}}_{j}\left(Q\right)-p_{ij}\left(P,Q\right){\dot{u}}_{j}\left(Q\right)\right]d\Gamma +\underset{\Omega }{\int }\sigma_{jki}^{\left[1\right]}\left(P,q\right){\dot{\varepsilon }}_{Ijk}^{\left[1\right]}\left(q\right)d\Omega \\ & +\underset{\Gamma }{\int }\left\{T\left(Q\right)\frac{\partial u_{i}^{T\left(1\right)}\left(P,Q\right)}{\partial n}-\frac{\partial T\left(Q\right)}{\partial n}u_{i}^{T\left(1\right)}\left(P,Q\right)\right\}d\Gamma \left(Q\right)\\ & +\lambda^{-1}\overset{2}{\underset{f=1}{\sum }}{\left(-1\right)}^{f}\underset{\Gamma }{\int }\left[\frac{\partial u_{i}^{Tff+1]}\left(P,Q\right)}{\partial n}W^{\left[f\right]}\left(Q\right)\right.\\ & \left.-u_{i}^{T\left(f+1\right)}\left(P,Q\right)\frac{\partial W^{\left(f\right]}\left(Q\right)}{\partial n}\right]d\Gamma +\lambda^{-1}\overset{M}{\underset{m=1}{\sum }}u_{i}^{T\left[3\right]A}\left(P,q_{m}\right)W^{\left[3\right]P}\left(q_{m}\right) \end{array}$$

where $c_{ij}$, ${\dot{\varepsilon }}_{Ijk}^{\left[1\right]}\left(q\right)$, ${\dot{u}}_{j}\left(Q\right)$, and ${\dot{p}}_{j}\left(Q\right)$ are the free coefficient, initial strain rate, displacement rate, and surface traction rate, respectively. However, r, Γ, and Ω are the distance between the observation point and loading point, the boundary, and domain, respectively.

According to [22], Kelvin’s solution $u_{ij}^{\left[1\right]}\left(p,q\right)$ and $p_{ij}\left(p,q\right)$ can be written as

(22) $$u_{ij}^{\left[1\right]}\left(p,q\right)=\frac{1}{16\pi \left(1-v\right)Gr}\left\{\left(3-4v\right)\delta_{ij}+r_{,i}r_{,j}\right\},\;r_{,i}=\partial r/\partial x_{i}$$

(23) $$p_{ij}\left(p,q\right)=\frac{1}{8\pi \left(1-v\right)Gr^{2}}\left\{\left[\left(1-2v\right)\delta_{ij}+3r_{,i}r_{,j}\right]\frac{\partial r}{\partial n}-\left(1-2v\right)\left(r_{,i}n_{j}-r_{,j}n_{i}\right)\right\},\;$$

The functions $\sigma_{ijk}^{\left[1\right]}\left(p,q\right),\;u_{i}^{T\left[f\right]}\left(p,q\right),\frac{\partial u_{i}^{T\left[f\right]}\left(p,q\right)}{\partial n}$, and $u_{i}^{T\left[3\right]A}\left(p,q\right)$ in Equation (21) can be expressed as [1,16]

(24) $$\sigma_{jki}^{\left[1\right]}\left(p,q\right)=\frac{-1}{8\pi \left(1-v\right)r^{2}}\left\{\left(1-2v\right)\left(\delta_{ji}r_{,k}+\delta_{ki}r_{,j}-\delta_{jk}r_{,i}\right)+3r_{,i}r_{,j}r_{,k}\right\},\;$$

(25) $$\;u_{i}^{T\left[f\right]}\left(p,q\right)=m_{0}T_{,i}^{f+1)}\left(p,q\right)=\frac{m_{0}\left(2f-1\right)r_{,i}r^{2f-2}}{4\pi \left(2f\right)!},\;m_{0}=\frac{\left(1+v\right)\alpha }{\left(1-v\right)}\;$$

(26) $$\frac{\partial \;u_{i}^{T\left[f\right]}\left(p,q\right)}{\partial n}=\frac{m_{0}\left(2f-1\right)r^{2f-3}}{4\pi \left(2f\right)!}\left[n_{i}+\left(2f-3\right)r_{,i}\;\frac{\partial r}{\partial n}\right],\;$$

(27) $$\begin{array}{ll} u_{i}^{T\left[3\right]A}\left(p,q\right) & =m_{0}T_{,i}^{\left[4\right]A}\left(p,q\right)\\ & =\frac{m_{0}A^{3}r_{,i}\left(105\;r^{6}+189\;r^{4}A^{2}+27\;r^{2}A^{4}-A^{6}\right)}{45360\;r^{2}},\;r>A \end{array}\;$$

(28) $$u_{i}^{T\left[3\right]A}\left(p,q\right)=\frac{m_{0}r\;r_{,i}\left(-r^{6}+27\;r^{4}A^{2}+189\;r^{2}A^{4}+105\;A^{6}\right)}{45360},\;\;r\leq A\;$$

where α denotes the thermal expansion coefficient.

Based on the initial stress formulation, the domain integral in Equation (21) can be written as [1]

(29) $$\Pi =\underset{\Omega }{\int }\varepsilon_{ijk}^{\left[1\right]}\left(P,q\right){\dot{\sigma }}_{Ijk}^{\left[1\right]}\left(q\right)d\Omega ,$$

where

(30) $$\varepsilon_{ijk}^{\left[1\right]}\left(p,q\right)=\left[\left(1-2v\right)\left(\delta_{ij}r_{,k}+\delta_{ik}r_{,j}\right)-\delta_{jk}r_{,i}+3\;r_{,i}\;r_{,j}\;r_{,k}\right]\frac{-1}{16\pi \left(1-v\right)Gr^{2}}.\;$$

The following equations are used for initial stress interpolation [8,9]:

(31) $$\nabla^{2}{\dot{\sigma }}_{Ijk}^{\left[1\right]S}\left(q\right)=-{\dot{\sigma }}_{Ijk}^{\left[2\right]S}\left(q\right),$$

(32) $$\nabla^{2}{\dot{\sigma }}_{Ijk}^{\left[2\right]S}\left(q\right)=-\overset{M}{\underset{m=1}{\sum }}{\dot{\sigma }}_{Ijk}^{\left[3\right]PA}\left(q_{m}\right),$$

The initial stress rate ${\dot{\sigma }}_{ljk}^{\left[2\right]s}\left(q\right)$ curvature can be expressed as

(33) $$\begin{array}{l} c{\dot{\sigma }}_{Ijk}^{\left[2\right]S}\left(P\right)=\underset{\Gamma }{\int }\left\{T^{\left[1\right]}\left(P,Q\right)\frac{\partial {\dot{\sigma }}_{Ijk}^{\left[2\right]S}\left(Q\right)}{\partial n}-\frac{\partial T^{\left[1\right]}\left(P,Q\right)}{\partial n}{\dot{\sigma }}_{Ijjk}^{\left[2\right]S}\left(Q\right)\right\}d\Gamma \\ +\overset{M}{\underset{m=1}{\sum }}T^{\left[1\right]A}\left(P,q_{m}\right){\dot{\sigma }}_{Ijk}^{\left[3\right]PA}\left(q_{m}\right) \end{array}$$

in which *M* is the number of points ${\dot{\sigma }}_{[jk}^{\left[3\right]PA}\left(q\right)$.

On the boundary, the initial stress rate ${\dot{\sigma }}_{Ijk}^{\left[1\right]}\left(P\right)$ can be written as

(34) $$\begin{array}{ll} c{\dot{\sigma }}_{Ijk}^{\left[1\right]S}\left(p\right)= & -\overset{2}{\underset{f=1}{\sum }}{\left(-1\right)}^{f}\int_{\Gamma }\left\{T^{\left[f\right]}\left(P,Q\right)\frac{\partial {\dot{\sigma }}_{Ijk}^{\left[f\right]S}\left(Q\right)}{\partial n}-\frac{\partial T^{\left[f\right]}\left(P,Q\right)}{\partial n}{\dot{\sigma }}_{Ijk}^{\left[f\right]}\left(Q\right)\right\}d\Gamma \\ & -\overset{M}{\underset{m=1}{\sum }}T^{\left[2\right]A}\left(P,q_{m}\right){\dot{\sigma }}_{Ijk}^{\left[3\right]PA}\left(q_{m}\right). \end{array}$$

For internal points, the following equation is obtained in the same manner as Equation (34)

(35) $$\begin{array}{c} c{\dot{\sigma }}_{Ijk}^{\left[1\right]S}\left(p\right)=-\overset{2}{\underset{f=1}{\sum }}{\left(-1\right)}^{f}\int_{\Gamma }\left\{T^{\left[f\right]}\left(p,Q\right)\frac{\partial {\dot{\sigma }}_{Ijk}^{[f|S}\left(Q\right)}{\partial n}-\frac{\partial T^{\left[f\right]}\left(p,Q\right)}{\partial n}{\dot{\sigma }}_{Ijk}^{\left[f\right]S}\left(Q\right)\right\}d\Gamma \\ -\overset{M}{\underset{m=1}{\sum }}T^{\left[2\right]A}\left(p,q_{m}\right){\dot{\sigma }}_{Ijk}^{\left[3\right]PA}\left(q_{m}\right). \end{array}$$

For performing the interpolation process, the following equations were employed [15]:

(36) $$\nabla^{2}{\dot{\varepsilon }}_{Ijk}^{\left[1\right]S}\left(q\right)=-{\dot{\varepsilon }}_{Ijk}^{\left[2\right]S}\left(q\right),\;$$

(37) $$\nabla^{2}{\dot{\varepsilon }}_{Ijk}^{\left[2\right]S}\left(q\right)=-\overset{M}{\underset{m=1}{\sum }}{\dot{\varepsilon }}_{Ijk}^{\left[3\right]PA}\left(q_{m}\right),$$

where $\nabla^{2}=\partial^{2}/\partial x^{2}+\partial^{2}/\partial y^{2}+\partial^{2}/\partial z^{2}$.

From Equations (36) and (37), the following equation is established:

(38) $$\nabla^{4}{\dot{\varepsilon }}_{Ijk}^{\left[1\right]S}\left(q\right)=\overset{M}{\underset{m=1}{\sum }}{\dot{\varepsilon }}_{Ijk}^{\left[3\right]PA}\left(q_{m}\right),$$

In this method, each initial strain component ${\dot{\varepsilon }}_{Ijk}^{\left[1\right]S}\left(q\right)\left(j,k=1,2,3\right)$ is interpolated.

Using the Green’s second identity and Equation (37), the following result is obtained [8,9]:

(39) $$\begin{array}{ll} C{\dot{\varepsilon }}_{Ijk}^{\left[2\right]S}\left(P\right)= & \underset{\Gamma }{\int }\left\{T^{\left[1\right]}\left(P,Q\right)\frac{\partial {\dot{\varepsilon }}_{Ijk}^{\left[2\right]S}\left(Q\right)}{\partial n}-\frac{\partial T^{\left[1\right]}\left(P,Q\right)}{\partial n}{\dot{\varepsilon }}_{Ijk}^{\left[2\right]S}\left(Q\right)\right\}d\Gamma \\ & +\overset{M}{\underset{m=1}{\sum }}T^{\left[1\right]A}\left(P,q_{m}\right){\dot{\varepsilon }}_{Ijk}^{\left[3\right]PA}\left(q_{m}\right). \end{array}$$

Now, using the Green’s theorem and Equations (36) and (37), the initial strain rate ${\dot{\varepsilon }}_{Ijk}^{\left[1\right]}\left(P\right)$ can be expressed as [7,8]

(40) $$\begin{array}{cc} C{\dot{\varepsilon }}_{Ijk}^{\left[1\right]S}\left(P\right)= & \underset{\Gamma }{\int }\left\{T^{\left[1\right]}\left(P,Q\right)\frac{\partial {\dot{\varepsilon }}_{Ijk}^{\left[1\right]S}\left(Q\right)}{\partial n}-\frac{\partial T^{\left[1\right]}\left(P,Q\right)}{\partial n}{\dot{\varepsilon }}_{Ijk}^{\left[1\right]S}\left(Q\right)\right\}d\Gamma \\ & +\underset{\Omega }{\int }T^{\left[2\right]}\left(P,q_{m}\right){\dot{\varepsilon }}_{Ijk}^{\left[2\right]}\left(q_{m}\right)\\ = & -\overset{2}{\underset{f=1}{\sum }}{\left(-1\right)}^{f}\underset{\Gamma }{\int }\left\{T^{\left[f\right]}\left(P,Q\right)\frac{\partial {\dot{\varepsilon }}_{Ijk}^{\left[f\right]}\left(Q\right)}{\partial n}-\frac{\partial T^{\left[f\right]}\left(P,Q\right)}{\partial n}{\dot{\varepsilon }}_{Ijk}^{\left[f\right]S}\left(Q\right)\right\}d\Gamma \\ & -\overset{M}{\underset{m=1}{\sum }}T^{\left[2\right]A}\left(P,q_{m}\right){\dot{\varepsilon }}_{Ijk}^{\left[3\right]PA}\left(q_{m}\right) \end{array}$$

where

$$C=\left\{\begin{array}{l} 0.5\;\mathrm{on}\;\mathrm{the}\;\mathrm{smooth}\;\mathrm{boundary}\\ 1\;\;\;\;\;\;\mathrm{in}\;\mathrm{the}\;\mathrm{domain} \end{array}\right.$$

It is assumed that ${\dot{\varepsilon }}_{Ijk}^{\left[2\right]S}\left(Q\right)$ is zero. For internal points, the following equation is obtained:

(41) $$\begin{array}{ll} c{\dot{\varepsilon }}_{Ijk}^{\left[1\right]S}\left(p\right)= & -\overset{2}{\underset{f=1}{\sum }}{\left(-1\right)}^{f}\int_{\Gamma }\left\{T^{\left[f\right]}\left(p,Q\right)\frac{\partial {\dot{\varepsilon }}_{Ijk}^{\left[f\right]S}\left(Q\right)}{\partial n}-\frac{\partial T^{\left[f\right]}\left(p,Q\right)}{\partial n}{\dot{\varepsilon }}_{Ijk}^{\left[j\right]S}\left(Q\right)\right\}d\Gamma \\ & -\overset{M}{\underset{m=1}{\sum }}T^{\left[2\right]A}\left(p,q_{m}\right){\dot{\varepsilon }}_{Ijk}^{\left[3\right]PA}\left(q_{m}\right). \end{array}$$

when the boundary is divided into $N_{0}$ constant elements and $N_{1}$ internal points, then $\left(2N_{0}+N_{1}\right)$ unknowns must be solved simultaneously.

The function $\sigma_{jki}^{\left[f\right]}\left(p,q\right)$ is defined as

(42) $$\nabla^{2}\sigma_{jki}^{\left[f+1\right]}\left(p,q\right)=\sigma_{jki}^{\left[f\right]}\left(p,q\right).\;$$

Using Equations (36), (37), and (42) and Green’s second identity, Equation (21) becomes

(43) $$\begin{array}{ll} c_{ij}\left(P\right){\dot{u}}_{j}\left(P\right)= & \underset{\Gamma }{\int }\left[u_{ij}^{\left[1\right]}\left(P,Q\right){\dot{p}}_{j}\left(Q\right)-p_{ij}\left(P,Q\right){\dot{u}}_{j}\left(Q\right)\right]d\Gamma \\ & -\overset{2}{\underset{f=1}{\sum }}{\left(-1\right)}^{f}\underset{\Gamma }{\int }\left\{\frac{\partial \sigma_{jki}^{\left[f+1\right]}\left(P,Q\right)}{\partial n}{\dot{\varepsilon }}_{Ijk}^{\left[j\right]}\left(Q\right)-\sigma_{jki}^{\left[f+1\right]}\left(P,Q\right)\frac{\partial {\dot{\varepsilon }}_{Ijk}^{\left[f\right]S}\left(Q\right)}{\partial n}\right\}d\Gamma \\ & +\overset{M}{\underset{m=1}{\sum }}\sigma_{jki}^{\left[3\right]A}\left(P,q\right){\dot{\varepsilon }}_{Ijk\left(m\right)]}^{\left[3\right]PA}\left(q\right)\\ & +\underset{\Gamma }{\int }\left\{T\left(Q\right)\frac{\partial u_{i}^{T\left[1\right]}\left(p,Q\right)}{\partial n}-\frac{\partial T\left(Q\right)}{\partial n}u_{i}^{T\left[1\right]}\left(p,Q\right)\right\}d\Gamma \left(Q\right)\\ & +\lambda^{-1}\overset{2}{\underset{f=1}{\sum }}{\left(-1\right)}^{f}\underset{\Gamma }{\int }\left[\frac{\partial u_{i}^{T\left[f+1\right]}\left(P,Q\right)}{\partial n}W^{\left[f\right]}\left(Q\right)\right.\\ & \left.-u_{i}^{T\left(f+1\right]}\left(P,Q\right)\frac{\partial W^{\left[f\right]}}{\partial n}\right]d\Gamma +\lambda^{-1}\overset{M}{\underset{m=1}{\sum }}u_{i}^{\left[3\right]A}\left(P,q_{m}\right)W^{\left[3\right]PA}\left(q_{m}\right). \end{array}$$

The Kelvin solutions $u_{ij}^{\left[f\right]}$ and $u_{ij}^{\left[f\right]A}$ can be expressed as [8,9]

(44) $$u_{ij}^{\left[f\right]}=\frac{-1}{2\left(1-v\right)G}T_{,ij}^{\left[f+1\right]}+\frac{\delta_{ij}T_{,kk}^{\left[f+1\right]}}{G}.\;$$

(45) $$u_{ij}^{\left[f\right]A}=\frac{-1}{2\left(1-v\right)G}T_{,ij}^{\left[f+1\right]A}+\frac{\delta_{ij}T_{,kk}^{\left[f+1\right]A}}{G}.\;$$

Equation (44) can be expressed using Equations (39), (40), and (45) as follows [9]:

(46) $$u_{ij}^{\left[f\right]}=\frac{\left(2f-1\right)r^{2f-3}}{8\pi \left(1-v\right)G\left(2f\right)!}\left[\left(4f-1-4fv\right)\delta_{ij}-\left(2f-3\right)r_{,i}r_{,j}\right],$$

(47) $$\begin{array}{ll} u_{ij}^{\left[3\right]A}= & \frac{-A^{3}}{90720\left(1-v\right)Gr^{3}}\left\{\delta_{ij}\left(105r^{6}+189A^{2}r^{4}+27A^{4}r^{2}-A^{6}\right)\right.\\ & +3r_{i,}r_{,j}\left(105r^{6}+63A^{2}r^{4}-9A^{4}r^{2}+A^{6}\right)\\ & \left.-36\left(1-v\right)\delta_{ij}r^{2}\left(35r^{4}+42A^{2}r^{2}+3A^{4}\right)\right\}\;r>A\;\left(A-9\right), \end{array}$$

(48) $$\begin{array}{ll} u_{ij}^{\left[3\right]A}= & \frac{-1}{90720\left(1-v\right)G}\left\{\delta_{ij}\left(-r^{6}+27A^{2}r^{4}+189A^{4}r^{2}+105A^{6}\right)\right.\\ & +6r_{,i}r_{,j}r^{2}\left(-r^{4}+18A^{2}r^{2}+63A^{4}\right)\\ & \left.-18\delta_{ij}\left(1-v\right)\left(-r^{6}+21A^{2}r^{4}+105A^{4}r^{2}+35A^{6}\right)\right\}\;\;r\leq A \end{array}$$

The function $\varepsilon_{jki}^{\left[f\right]}\left(p,q\right)$ is described as follows:

(49) $$\nabla^{2}\varepsilon_{jki}^{\left[f+1\right]}\left(p,q\right)=\varepsilon_{jki}^{\left[f\right]}\left(p,q\right)$$

The domain integral in (28) can be expressed as

(50) $$\begin{array}{ll} \Pi = & -\overset{2}{\underset{f=1}{\sum }}{\left(-1\right)}^{f}\underset{\Gamma }{\int }\left\{\frac{\partial \varepsilon_{jki}^{\left[f+1\right]}\left(P,Q\right)}{\partial n}{\dot{\sigma }}_{ljk}^{\left[f\right]S}\left(Q\right)-\varepsilon_{jki}^{\left[f+1\right]}\left(P,Q\right)\frac{\partial {\dot{\sigma }}_{ljk}^{\left[f\right]S}\left(Q\right)}{\partial n}\right\}d\Gamma \\ & +\overset{M}{\underset{m=1}{\sum }}\varepsilon_{jki}^{[3kA}\left(P,q_{m}\right){\dot{\sigma }}_{ljk}^{\left[3\right]RA}\left(q_{m}\right) \end{array}$$

Using Equation (46), $\varepsilon_{ijk}^{\left[f\right]}\left(p,q\right)$ is obtained as

(51) $$\begin{array}{cc} \varepsilon_{ijk}^{\left[f\right]}\left(p,q\right)= & \frac{\partial u_{ij}^{\left[f\right]}}{\partial x_{k}}+\frac{\partial u_{kj}^{\left[f\right]}}{\partial x_{i}}\\ = & \frac{\left(2f-1\right)\left(2f-3\right)r^{2f-4}}{8\pi \left(1-v\right)\left(2f\right)!G}\left[\left(2f-1-2fv\right)\left(\delta_{jk}r_{,i}+\delta_{ik}r_{,j}\right)\right.\\ & \left.-\delta_{ij}r_{,k}-\left(2f-5\right)r_{,i}r_{,j}r_{,k}\right] \end{array}$$

Furthermore, using Equations (47) and (48), the normal derivatives $\partial \varepsilon_{ijk}^{\left[f\right]}\left(p,q\right)/\partial n$ and $\varepsilon_{ijk}^{\left[3\right]A}\left(p,q\right)$ are obtained as

(52) $$\begin{array}{l} \frac{\partial \varepsilon_{ijk}^{\left[f\right]}\left(p,q\right)}{\partial n}=\frac{\left(2f-1\right)\left(2f-3\right)r^{2f-5}}{8\pi \left(1-v\right)\left(2f\right)!G}\left\{\left(2f-5\right)\left[\left(2f-1-2fv\right)\left(\delta_{jk}r_{,i}+\delta_{ik}r_{,j}\right)\right.\right.\\ \left.-\delta_{ij}r_{,k}-\left(2f-7\right)r_{,i}r_{,j}r_{,k}\right]\frac{\partial r}{\partial n}-\left(2f-5\right)\left(r_{,j}r_{,k}n_{i}+r_{,i}r_{,k}n_{j}+r_{,i}r_{,j}n_{k}\right)\\ \left.+\left(2f-1-2fv\right)\left(\delta_{jk}n_{i}+\delta_{ik}n_{j}\right)-\delta_{ij}n_{k}\right\}, \end{array}$$

(53) $$\begin{array}{cc} \varepsilon_{ijk}^{\left[3\right]A}\left(p,q\right)= & \frac{\partial u_{ij}^{\left[3\right]A}}{\partial x_{k}}+\frac{\partial u_{kj}^{\left[3\right]A}}{\partial x_{i}}\\ = & \frac{A^{3}}{30240\left(1-v\right)r^{4}G}[-\left(\delta_{jk}r_{,i}+\delta_{ik}r_{,j}+\delta_{ij}r_{,k}\right)(105r^{6}\\ & +63A^{2}r^{4}-9A^{4}r^{2}+A^{6})-r_{,i}r_{,j}r_{,k}(105r^{6}-63A^{2}r^{4}\\ & +27A^{4}r^{2}-5A^{6})+18\left(1-v\right)\left(\delta_{jk}r_{,i}+\delta_{ik}r_{,j}\right)\\ & \times r^{2}\left(35r^{4}+14A^{2}r^{2}-A^{4}\right)]\;r>A, \end{array}$$

(54) $$\begin{array}{cc} \varepsilon_{ijk}^{\left[3\right]A}\left(p,q\right)= & \frac{r}{15120\left(1-v\right)G}\left[-\left(\delta_{jk}r_{,i}+\delta_{ik}r_{,j}+\delta_{ij}r_{,k}\right)\left(-r^{4}+18A^{2}r^{2}+63A^{4}\right)\right.\\ & -4r_{,i}r_{j}r_{,k}r^{2}\left(-r^{2}+9A^{2}\right)+9\left(1-v\right)\left(\delta_{jk}r_{,i}+\delta_{ik}r_{,j}\right)\\ & \left.\times \left(-r^{4}+14A^{2}r^{2}+35A^{4}\right)\right]\;r\leq A, \end{array}$$

Using the stress–strain relationship, $\sigma_{ijk}^{\left[f\right]}\left(p,q\right)$ is obtained as

(55) $$\begin{array}{cc} \sigma_{ijk}^{\left[f\right]}\left(p,q\right)= & \frac{2vG}{1-2v}\delta_{ik}\frac{\partial u_{mj}^{\left[f\right]}}{\partial x_{m}}+G\left[\frac{\partial u_{ij}^{\left[f\right]}}{\partial x_{k}}+\frac{\partial u_{kj}^{\left[f\right]}}{\partial x_{i}}\right]\\ = & \frac{\left(2f-1\right)\left(2f-3\right)r^{2f-4}}{4\pi \left(1-v\right)\left(2f\right)!}\left\{\left(2f-1-2fv\right)\left(\delta_{jk}r_{,i}+\delta_{ik}r_{,j}\right)\right.\\ & \left.-\left(1-2fv\right)\delta_{ij}r_{,k}-\left(2f-5\right)\;r_{,i}\;r_{,j}\;r_{,k}\right\}. \end{array}$$

Moreover, the normal derivatives $\partial \sigma_{ijk}^{\left[f\right]}\left(p,q\right)/\partial n$ and $\sigma_{ijk}^{\left[3\right]A}\left(p,q\right)$ are given by [11]

(56) $$\begin{array}{cc} \frac{\partial \sigma_{ijk}^{\left[f\right]}\left(p,q\right)}{\partial n}= & \frac{\left(2f-1\right)\left(2f-3\right)}{4\pi \left(1-v\right)\left(2f\right)!}r^{2f-5}\left[\left(2f-5\right)\left\{\left(2f-1-2fv\right)\left(\delta_{jk}r_{,i}+\delta_{ik}r_{,j}\right)\right.\right.\\ & \left.-\left(1-2fv\right)\delta_{ij}r_{,k}-\left(2f-7\right)r_{,i}r_{,j}r_{,k}\right\}\frac{\partial r}{\partial n}\\ & -\left(2f-5\right)\left(r_{,j}r_{,k}n_{i}+r_{,i}r_{,k}n_{j}+r_{,i}r_{,j}n_{k}\right)\\ & \left.+\left(2f-1-2fv\right)\left(\delta_{jk}n_{i}+\delta_{ik}n_{j}\right)-\left(1-2fv\right)\delta_{ij}n_{k}\right], \end{array}$$

(57) $$\begin{array}{cc} \sigma_{ijk}^{\left[3\right]A}\left(p,q\right)= & \frac{A^{3}}{15120\left(1-v\right)r^{4}}\{18v\delta_{ij}r_{,k}r^{2}\left(35\;r^{4}+14\;A^{2}r^{2}-A^{4}\right)\\ & -\left(\delta_{jk}r_{,i}+\delta_{ik}r_{,j}+\delta_{ij}r_{,k}\right)\left(105\;r^{6}+63\;A^{2}r^{4}-9\;A^{4}r^{2}+A^{6}\right)\\ & -r_{i,}r_{,j}r_{,k}\left(105\;r^{6}-63\;A^{2}r^{4}+27\;A^{4}r^{2}-5\;A^{6}\right)\\ & +18\left(1-v\right)\left(\delta_{jk}r_{,i}+\delta_{ik}r_{,j}\right)r^{2}\left(35\;r^{4}+14\;A^{2}r^{2}-A^{4}\right)\}\;r>A, \end{array}$$

(58) $$\begin{array}{cc} \sigma_{ijk}^{\left[3\right]A}\left(p,q\right)= & \frac{r}{7560\left(1-v\right)}\left\{9v\delta_{ij}r_{,k}\left(-r^{4}+14\;A^{2}r^{2}+35\;A^{4}\right)\right.\\ & -\left(\delta_{jk}r_{,i}+\delta_{ik}r_{,j}+\delta_{ij}r_{,k}\right)\left(-r^{4}+18\;A^{2}r^{2}+63\;A^{4}\right)\\ & -4r_{,i}r_{,j}r_{,k}r^{2}\left(-r^{2}+9\;A^{2}\right)+9\left(1-v\right)\left(\delta_{jk}r_{,i}+\delta_{ik}r_{,j}\right)\\ & \left.\times \left(-r^{4}+14\;A^{2}r^{2}+35\;A^{4}\right)\right\}\;\;r\leq A. \end{array}\;$$

The internal stress is given by [22]

(59) $$\begin{array}{ll} {\dot{\sigma }}_{ij}\left(p\right)= & \underset{\Gamma }{\int }\left[-\sigma_{kij}^{\left[1\right]}\left(p,Q\right){\dot{p}}_{k}\left(Q\right)-S_{kij}\left(p,Q\right){\dot{u}}_{k}\left(Q\right)\right]d\Gamma +\underset{\Omega }{\int }\sigma_{ijks}^{\left[1\right]}\left(p,q\right){\dot{\varepsilon }}_{Iks}^{\left[1\right]}\left(q\right)d\Omega \\ & -{\dot{\sigma }}_{Iij}^{\left[1\right]}\left(p\right)+\underset{\Gamma }{\int }\left[\frac{\partial \sigma_{ij}^{T\left[1\right]}\left(p,Q\right)}{\partial n}\dot{T}\left(Q\right)-\sigma_{ij}^{T\left[1\right]}\left(p,Q\right)\frac{\partial \dot{T}\left(Q\right)}{\partial n}\right]d\Gamma \\ & +\lambda^{-1}\overset{2}{\underset{f=1}{\sum }}{\left(-1\right)}^{f}\underset{\Gamma }{\int }\left[\frac{\partial \sigma_{ij}^{T\left[f+1\right]}\left(p,Q\right)}{\partial n}W^{\left[f\right]}\left(Q\right)\right.\\ & \left.-\sigma_{ij}^{T\left[f+1\right]}\left(p,Q\right)\frac{\partial W^{\left[f\right]}\left(Q\right)}{\partial n}\right]d\Gamma +\lambda^{-1}\overset{M}{\underset{m=1}{\sum }}\sigma_{ij}^{\left[3\right]A}\left(P,q_{m}\right)W^{\left[3\right]PA}\left(q_{m}\right), \end{array}$$

where ${\dot{\sigma }}_{\mathrm{Iij}\;}^{\left[1\right]}\left(p\right)$ represents the initial stress derived from the initial strain. Additionally, $S_{kij}\left(p,q\right)$ and $\sigma_{ijks}^{\left[1\right]}\left(p,q\right)$ in Equation (36) can be expressed as [1,11]

(60) $$\begin{array}{ll} S_{kij}\left(p,q\right)= & \frac{G}{4\pi \left(1-v\right)r^{3}}\left\{3\frac{\partial r}{\partial n}\left[\left(1-2v\right)\delta_{ij}r_{,k}+v\left(\delta_{ik}r_{,j}+\delta_{jk}r_{,i}\right)-5r_{,i}r_{,j}r_{,k}\right]\right.\\ & +3v\left(n_{i}r_{,j}r_{,k}+n_{j}r_{,i}r_{,k}\right)+\left(1-2v\right)\left(3n_{k}r_{,i}r_{,j}+n_{j}\delta_{ik}+n_{i}\delta_{jk}\right)\\ & \left.-\left(1-4v\right)n_{k}\delta_{ij}\right\}, \end{array}\;$$

(61) $$\begin{array}{ll} \sigma_{ijkl}^{\left[1\right]}\left(p,q\right)= & \frac{1}{4\pi \left(1-v\right)r^{3}}\left[3\left(1-2v\right)\left(\delta_{ij}r_{,k}r_{,l}+\delta_{kl}r_{,i}r_{,j}\right)+3v\left(\delta_{il}r_{,j}r_{,k}\right.\right.\\ & \left.+\delta_{jk}r_{,i}r_{,l}+\delta_{ik}r_{,j}r_{,s}+\delta_{jl}r_{,i}r_{,k}\right)+\left(1-2v\right)\left(\delta_{ik}\delta_{lj}+\delta_{jk}\delta_{li}\right)\\ & \left.-\left(1-4v\right)\delta_{ij}\delta_{kl}-15\;r_{,i}r_{,j}r_{,k}r_{,l}\right], \end{array}$$

(62) $$\begin{array}{ll} \sigma_{ij}^{T\left(f\right]}\left(p,q\right) & =2Gm_{0}\left[\frac{\partial^{2}T^{\left[f+1\right]}}{\partial x_{i}\partial x_{j}}-\delta_{ij}T^{\left[f\right]}\right]\\ & =\frac{Gm_{0}\left(2f-1\right)r^{2f-3}}{2\pi \left(2f\right)!}\left[-\left(2f-1\right)\delta_{ij}+\left(2f-3\right)r_{,i}r_{,j}\right], \end{array}$$

(63) $$\begin{array}{c} \frac{\partial \sigma_{ij}^{Tff}\left(p,q\right)}{\partial n}=\frac{Gm_{0}\left(2f-1\right)r^{2f-4}}{2\pi \left(2f\right)!}\left[r_{,j}n_{i}+r_{,i}n_{,j}-\left(2f-1\right)\frac{\partial r}{\partial n}\delta_{ij}\right.\\ \;\;\;\;\;\left.+\left(2f-5\right)r_{,i}r_{,j}\frac{\partial r}{\partial n}\right], \end{array}\;$$

(64) $$\begin{array}{cc} \sigma_{ij}^{T\left[3\right]A}\left(p,q\right)= & 2Gm_{0}\left[\frac{\partial^{2}T^{\left[4\right]A}}{\partial x_{i}\partial x_{j}}-\delta_{ij}T^{[3\}A}\right]\\ = & \frac{Gm_{0}A^{3}}{22680r^{3}}\left[-\delta_{ij}\left(525r^{6}+567r^{4}A^{2}+27r^{2}A^{4}+A^{6}\right)\right.\\ & \left.+3\left(105r^{6}+63r^{4}A^{2}-9r^{2}A^{4}+A^{6}\right)r_{,i}r_{j}\right]\;\;r>A, \end{array}$$

(65) $$\begin{array}{ll} \sigma_{ij}^{T\left[3\right]A}\left(p,q\right)= & \frac{Gm_{0}}{11340}\left[\delta_{ij}\left(4r^{6}-81r^{4}A^{2}-378r^{2}A^{4}-105A^{6}\right)\right.\\ & \left.+3r^{2}\left(-r^{4}+18r^{2}A^{2}+63A^{4}\right)r_{,i}r_{,j}\right]\;\;r≦A, \end{array}$$

The function $\sigma_{ijkl}^{\left[f\right]}\left(p,q\right)$ is defined as

(66) $$\nabla^{2}\sigma_{ijkl}^{\left[f+1\right]}\left(p,q\right)=\sigma_{ijkl}^{\left[f\right]}\left(p,q\right).\;$$

Using Green’s theory and Equation (66), Equation (59) can be written as

(67) $$\begin{array}{ll} {\dot{\sigma }}_{ij}\left(p\right)= & \underset{\Gamma }{\int }\left[-\sigma_{kij}^{\left[1\right]}\left(p,Q\right){\dot{p}}_{k}\left(Q\right)-S_{kij}\left(p,Q\right){\dot{u}}_{k}\left(Q\right)\right]d\Gamma \\ & -\overset{2}{\underset{f=1}{\sum }}{\left(-1\right)}^{f}\underset{\Gamma }{\int }\left[\frac{\partial \sigma_{ijkl}^{\left[f+1\right]}\left(p,Q\right)}{\partial n}{\dot{\varepsilon }}_{Ikl}^{fj]S}\left(Q\right)-\sigma_{ijkl}^{\left[f+1\right]}\left(p,Q\right)\frac{\partial {\dot{\varepsilon }}_{Ikl}^{\left[f\right]S}\left(Q\right)}{\partial n}\right]d\Gamma \\ & +\overset{M}{\underset{m=1}{\sum }}\sigma_{ijkl}^{\left[3\right]A}\left(p,q_{m}\right){\dot{\varepsilon }}_{Ikl}^{\left[3\right]PA}\left(q_{m}\right)-{\dot{\sigma }}_{Iij}^{\left[1\right]}\left(p\right)\\ & +\underset{\Gamma }{\int }\left[\frac{\partial \sigma_{ij}^{T\left[1\right]}\left(p,Q\right)}{\partial n}\dot{T}\left(Q\right)-\sigma_{ij}^{T\left[1\right]}\left(p,Q\right)\frac{\partial \dot{T}\left(Q\right)}{\partial n}\right]d\Gamma \\ & +\lambda^{-1}\overset{2}{\underset{f=1}{\sum }}{\left(-1\right)}^{f}\underset{\Gamma }{\int }\left[\frac{\partial \sigma_{ij}^{Tff+1]}\left(p,Q\right)}{\partial n}W^{\left[f\right]}\left(Q\right)\right.\\ & \left.-\sigma_{ij}^{T\left[f+1\right]}\left(p,Q\right)\frac{\partial W^{\left[f\right]}\left(Q\right)}{\partial n}\right]d\Gamma +\lambda^{-1}\overset{M}{\underset{m=1}{\sum }}\sigma_{ij}^{\left[3\right]A}\left(P,q_{m}\right)W^{\left[3\right]PA}\left(q_{m}\right). \end{array}$$

Using Equation (55) and the relationship between displacement and stress, $\sigma_{ijkl}^{\left[f\right]}\left(p,q\right)$ is obtained as

(68) $$\begin{array}{cc} \sigma_{ijkl}^{\left[f\right]}\left(p,q\right)= & \frac{2vG}{\left(1-2v\right)}\delta_{ij}\sigma_{mkl,m}^{\left[f\right]}\left(p,q\right)+G\left[\sigma_{ikl,j}^{\left[f\right]}\left(p,q\right)+\sigma_{jkl,i}^{fj}\left(p,q\right)\right]\\ = & \frac{\left(2f-1\right)\left(2f-3\right)Gr^{2f-5}}{2\pi \left(1-v\right)\left(1-2v\right)\left(2f\right)!}<2fv\left\{1+2\left(f-2\right)v\right\}\delta_{ij}\delta_{kl}+(1-2v\\ & \times \left[\left(2f-1-2fv\right)\left(\delta_{ik}\delta_{jl}+\delta_{il}\delta_{jk}\right)+\left(2f-5\right)\left(f-1-fv\right)\left(\delta_{jl}r_{,i}r_{,k}\right.\right.\\ & \left.+\delta_{jk}r_{,i}r_{,l}+\delta_{il}r_{,j}r_{,k}+\delta_{ik}r_{,j}r_{,l}\right)-\left(1-2fv\right)\{\left(2f-5\right)\\ & \left.\left.\times \left(\delta_{kl}r_{,i}r_{,j}+\delta_{ij}r_{,k}r_{,l}\right)+\delta_{ij}\delta_{kl}\right\}-\left(2f-5\right)\left(2f-7\right)r_{,i}r_{,j}r_{,k}r_{,l}\right]. \end{array}$$

Similarly, $\partial \sigma_{ijkl}^{\left[5\right]}\left(p,q\right)/\partial n$ and $\sigma_{ijkl}^{\left[3\right]A}\left(p,q\right)$ are obtained as

(69) $$\begin{array}{ll} \frac{\partial \sigma_{ijkl}^{ffl}\left(p,q\right)}{\partial n}= & \langle \langle \langle 2fv\left[1+2\left(f-2\right)v\right]\delta_{ij}\delta_{kl}+\left(1-2v\right)\{\left(2f-1-2fv\right)\left(\delta_{ik}\delta_{jl}+\delta_{il}\delta_{jk}\right)\\ & +\left(2f-7\right)\left(f-1-fv\right)\left(\delta_{jl}r_{,i}r_{,k}+\delta_{jk}r_{,i}r_{,l}+\delta_{il}r_{,j}r_{,k}+\delta_{ik}r_{,j}r_{,l}\right)\\ & -\left(1-2fv\right)\left[\left(2f-7\right)\left(\delta_{kl}r_{,i}r_{,j}+\delta_{ij}r_{,k}r_{,l}\right)+\delta_{ij}\delta_{kl}\right]\\ & -\left(2f-7\right)\left(2f-9\right)r_{,i}r_{,j}r_{,k}r_{,l}\}\rangle \frac{\partial r}{\partial n}+\left(1-2v\right)\\ & \times \{\left(f-1-fv\right)[\left(\delta_{jl}n_{k}+\delta_{jk}n_{l}\right)r_{,i}\\ & +\left(\delta_{il}n_{k}+\delta_{ik}n_{l}\right)r_{,j}+\left(\delta_{jl}n_{i}+\delta_{il}n_{j}\right)r_{,k}+\left(\delta_{jk}n_{i}+\delta_{ik}n_{j}\right)r_{,l}]\\ & -\left(1-2fv\right)\left[\delta_{ij}\left(r_{,l}n_{k}+r_{,k}n_{l}\right)+\delta_{kl}\left(r_{,j}n_{i}+r_{,i}n_{j}\right)\right]\\ & -\left(2f-7\right)\{\left(r_{,l}n_{,k}+r_{,k}n_{l}\right)r_{,i}r_{,j}+\left(r_{j}n_{i}+r_{,i}n_{j}\right)r_{,k}r_{,l}]\}\rangle \rangle , \end{array}$$

(70) $$\begin{array}{cc} \sigma_{ijkl}^{\left[3\right]A}\left(p,q\right)= & \frac{2vG}{\left(1-2v\right)}\delta_{ij}\sigma_{mkl,m}^{\left[3\right]A}\left(p,q\right)+G\left[\sigma_{ikl,j}^{\left[3\right]A}\left(p,q\right)+\sigma_{jkl,i}^{\left[3\right]A}\left(p,q\right)\right]\\ = & \frac{GA^{3}}{7560\left(1-v\right)\left(1-2v\right)r^{5}}\langle 18vr^{2}\delta_{kl}\{28vr^{2}\delta_{ij}\left(5r^{2}+A^{2}\right)\\ & +\left(1-2v\right)[\delta_{ij}\left(35r^{4}+14A^{2}r^{2}-A^{4}\right)\\ & +r_{,i}r_{,j}\left(35r^{4}-14A^{2}r^{2}+3A^{4}\right)]\}+\left(1-2v\right)\\ & \times \{18vr^{2}\delta_{ij}[\delta_{kl}\left(35r^{4}+14A^{2}r^{2}-A^{4}\right)\\ & +r_{,k}r_{,l}\left(35r^{4}-14A^{2}r^{2}+3A^{4}\right)]\\ & -\left(\delta_{ij}\delta_{kl}+\delta_{kj}\delta_{li}+\delta_{ki}\delta_{li}\right)\left(105r^{6}+63A^{2}r^{4}-9A^{4}r^{2}+A^{6}\right)\\ & -(\delta_{ij}r_{,k}r_{,l}+\delta_{kj}r_{,i}r_{,l}+\delta_{ki}r_{,j}r_{,l}\\ & +\delta_{kl}r_{,i}r_{,j}+\delta_{il}r_{,k}r_{j}+\delta_{jl}r_{,k}r_{,i})\left(105r^{6}-63A^{2}r^{4}+27A^{4}r^{2}-5A^{6}\right)\\ & -r_{j}r_{,i}r_{,k}r_{,l}\left(-105r^{6}+189A^{2}r^{4}-135A^{4}r^{2}+35A^{6}\right)\\ & +9\left(1-v\right)r^{2}[2\left(\delta_{ki}\delta_{jl}+\delta_{kj}\delta_{il}\right)\left(35r^{4}+14A^{2}r^{2}-A^{4}\right)\\ & +\left(\delta_{ki}r_{,j}r_{,l}+\delta_{kj}r_{,i}r_{,l}+\delta_{li}r_{,j}r_{,k}+\delta_{lj}r_{,i}r_{,k}\right)\\ & \times \left(35r^{4}-14A^{2}r^{2}+3A^{4}\right)]\rangle r>\;A, \end{array}$$

(71) $$\begin{array}{ll} \sigma_{ijkl}^{\left[3\right]A}\left(p,q\right)= & \frac{G}{3780\left(1-v\right)\left(1-2v\right)}<63v^{2}\delta_{ij}\delta_{kl}\left(-r^{4}+10A^{2}r^{2}+15A^{4}\right)\\ & +\left(1-2v\right)\{18v[\delta_{ij}\delta_{kl}\left(-r^{4}+14A^{2}r^{2}+35A^{4}\right)\\ & +2\left(\delta_{kl}r_{,i}r_{,j}+\delta_{ij}r_{,k}r_{,l}r^{2}\left(-r^{2}+7A^{2}\right)\right]\\ & -\left(\delta_{ij}\delta_{kl}+\delta_{ik}\delta_{jl}+\delta_{il}\delta_{jk}\right)\left(-r^{4}+18A^{2}r^{2}+63A^{4}\right)\\ & -4(\delta_{kl}r_{,i}r_{,j}+\delta_{jl}r_{,i}r_{,k}+\delta_{jk}r_{,i}r_{,l}+\delta_{il}r_{,j}r_{,k}+\delta_{ik}r_{,j}r_{,l}\\ & +\delta_{ij}r_{,k}r_{,l}r^{2}\left(-r^{2}+9A^{2}\right)\\ & +8r_{,i}r_{,j}r_{,k}r_{,l}r^{4}+9\left(1-v\right)[\left(\delta_{ik}\delta_{jl}+\delta_{il}\delta_{jk}\right)\left(-r^{4}+14A^{2}r^{2}+35A^{4}\right)\\ & +2\left(\delta_{jl}r_{,i}r_{,k}+\delta_{jk}r_{,i}r_{,l}+\delta_{il}r_{,j}r_{,k}+\delta_{ik}r_{,j}r_{,l}\right)r^{2}\left(-r^{2}+7A^{2}\right)]\}\;>r\leq A, \end{array}$$

According to [17], Equation (67) can be written in the following form:

(72) $$\begin{array}{ll} {\dot{\sigma }}_{ij}\left(p\right)= & \underset{\Gamma }{\int }\left[-\sigma_{kij}^{\left[1\right]}\left(p,Q\right){\dot{p}}_{k}\left(Q\right)-S_{kij}\left\{{\dot{u}}_{k}\left(Q\right)-{\dot{u}}_{k}\left(Q_{A}\right)-\alpha \left(p-Q_{A}\right)\dot{T}\left(Q_{A}\right)\right\}\right]d\Gamma \\ & +\underset{\Gamma }{\int }\left[\frac{\partial \sigma_{ij}^{T\left[1\right]}\left(p,Q\right)}{\partial n}\left\{\dot{T}\left(Q\right)-\dot{T}\left(Q_{A}\right)\right\}-\sigma_{ij}^{T\left[1\right]}\left(p,Q\right)\frac{\partial \dot{T}\left(Q\right)}{\partial n}\right]d\Gamma \\ & +\lambda^{-1}\overset{2}{\underset{f=1}{\sum }}{\left(-1\right)}^{f}\underset{\Gamma }{\int }\left[\frac{\partial \sigma_{ij}^{T\left[f+1\right]}\left(p,Q\right)}{\partial n}W^{\left[f\right]}\left(Q\right)\right.\\ & \left.-\sigma_{ij}^{T\left[f+1\right]}\left(p,Q\right)\frac{\partial W^{\left[f\right]}\left(Q\right)}{\partial n}\right]d\Gamma +\lambda^{-1}\overset{M}{\underset{m=1}{\sum }}\sigma_{ij}^{T\left[3\right]A}\left(P,q\right)W_{\left(m\right)}^{\left[3\right]PA}\left(q\right)\\ & +\overset{2}{\underset{f=1}{\sum }}{\left(-1\right)}^{f}\underset{\Gamma }{\int }\left[\frac{\partial \sigma_{ijks}^{\left[f+1\right]}\left(p,Q\right)}{\partial n}{\dot{\varepsilon }}_{Iks}^{\left[f\right]S}\left(Q\right)-\sigma_{ijks}^{\left[f+1\right]}\left(p,Q\right)\frac{\partial {\dot{\varepsilon }}_{Iks}^{\left[f\right]S}\left(Q\right)}{\partial n}\right]d\Gamma \\ & +\overset{M}{\underset{m=1}{\sum }}\sigma_{ijks}^{\left[3\right]A}\left(p,q_{m}\right){\dot{\varepsilon }}_{Iks}^{\left[3\right]PA}\left(q_{m}\right)-{\dot{\sigma }}_{Iij}^{\left[1\right]}\left(p\right) \end{array}$$

$\varepsilon_{ijkl}^{\left[f\right]}\left(p,q\right)$ is calculated using Equation (51) and the displacement–stress relationship as

(73) $$\begin{array}{cc} \varepsilon_{ijkl}^{\left[f\right]}\left(p,q\right)= & \frac{2vG}{\left(1-2v\right)}\delta_{ij}\varepsilon_{mkl,m}^{\left[f\right]}\left(p,q\right)+G\left[\varepsilon_{ikl,j}^{\left[f\right]}\left(p,q\right)+\varepsilon_{jkl,i}^{\left[f\right]}\left(p,q\right)\right]\\ = & \frac{\left(2f-1\right)\left(2f-3\right)r^{2f-5}}{4\pi \left(1-v\right)\left(2f\right)!}\{\left(2f-1-2fv\right)\left(\delta_{ik}\delta_{jl}+\delta_{il}\delta_{jk}\right)\\ & +\left(2f-5\right)\left(f-1-fv\right)\left(\delta_{jl}r_{,i}r_{,k}+\delta_{jk}r_{,i}r_{,l}+\delta_{il}r_{,j}r_{,k}+\delta_{ik}r_{,j}r_{,l}\right)\\ & -\left(1-2fv\right)\left[\left(2f-5\right)\delta_{kl}r_{,i}r_{,j}+\delta_{ij}\delta_{kl}\right]+\left(2f-5\right)\delta_{ij}r_{,k}r_{,l}\\ & -\left(2f-5\right)\left(2f-7\right)r_{,i}r_{,j}r_{,k}r_{,l}\}. \end{array}$$

$\partial \varepsilon_{ijkl}^{\left[f\right]}\left(p,q\right)/\partial n$ and $\varepsilon_{ijkl}^{\left[3\right]A}\left(p,q\right)$ are also obtained as

(74) $$\begin{array}{ll} \frac{\partial \varepsilon_{ijkl}^{\left[f\right]}\left(p,q\right)}{\partial n}= & \frac{\left(2f-1\right)\left(2f-3\right)\left(2f-5\right)r^{2f-6}}{2\pi \left(1-v\right)\left(2f\right)!}\langle [\left(2f-1-2fv\right)\left(\delta_{ik}\delta_{jl}+\delta_{il}\delta_{jk}\right)\\ & +\left(2f-7\right)\left(f-1-fv\right)\left(\delta_{jl}r_{,i}r_{,k}+\delta_{jk}r_{i}r_{,l}+\delta_{il}r_{,j}r_{,k}+\delta_{ik}r_{,j}r_{,l}\right)\\ & -\left(1-2fv\right)\left\{\left(2f-7\right)\left(\delta_{kl}r_{,i}r_{j}+\delta_{ij}r_{,k}r_{,l}\right)+\delta_{ij}\delta_{kl}\right\}-\left(2f-7\right)\\ & \times \left(2f-9\right)r_{,i}r_{,j}r_{,k}r_{,l}\frac{\partial r}{\partial n}\\ & +\left(f-1-fv\right)\{\left(\delta_{jl}n_{k}+\delta_{jk}n_{l}\right)r_{,i}+\left(\delta_{il}n_{k}+\delta_{ik}n_{l}\right)r_{j}\\ & +\left(\delta_{jl}n_{i}+\delta_{il}n_{j}\right)r_{,k}+\left(\delta_{jk}n_{i}+\delta_{ik}n_{j}\right)r_{,l}\}\\ & -\left(1-2fv\right)\left\{\delta_{ij}\left(r_{,l}n_{k}+r_{,k}n_{l}\right)+\delta_{kl}\left(r_{,j}n_{i}+r_{,i}n_{j}\right)\right\}\\ & -\left(2f-7\right)\{\left(r_{,i}n_{k}+r_{,k}n_{l}\right)r_{,i}r_{j}+\left(r_{,j}n_{i}+r_{,i}n_{j}\right)r_{,k}r_{,l}\}\rangle , \end{array}$$

(75) $$\begin{array}{cc} \varepsilon_{ijkl}^{\left[3\right]A}\left(p,q\right)= & \frac{2vG}{\left(1-2v\right)}\delta_{ij}\varepsilon_{mkl,m}^{\left[3\right]A}\left(p,q\right)+G\left[\varepsilon_{ikl,j}^{\left[3\right]A}\left(p,q\right)+\varepsilon_{jkl,i}^{\left[3\right]A}\left(p,q\right)\right]\\ = & \frac{A^{3}}{15120\left(1-v\right)r^{5}}[18vr^{2}\delta_{ij}\{\delta_{kl}\left(35r^{4}+14A^{2}r^{2}-A^{4}\right)\\ & +r_{,k}r_{,l}\left(35r^{4}-14A^{2}r^{2}+3A^{4}\right)\}\\ & -\left(\delta_{ij}\delta_{kl}+\delta_{kj}\delta_{li}+\delta_{ki}\delta_{lj}\right)\left(105r^{6}+63A^{2}r^{4}-9A^{4}r^{2}+A^{6}\right)\\ & -(\delta_{ij}r_{,k}r_{,l}+\delta_{kj}r_{,i}r_{,l}+\delta_{ki}r_{,j}r_{,l}\\ & +\delta_{kl}r_{,}r_{j}+\delta_{il}r_{,k}r_{,j}+\delta_{jl}r_{,k}r_{,i})\left(105r^{6}-63A^{2}r^{4}+27A^{4}r^{2}-5A^{6}\right)\\ & -r_{j}r_{,i}r_{,k}r_{,l}\left(-105r^{6}+189A^{2}r^{4}-135A^{4}r^{2}+35A^{6}\right)\\ & +9\left(1-v\right)r^{2}\{2\left(\delta_{ki}\delta_{jl}+\delta_{kj}\delta_{il}\right)\left(35r^{4}+14A^{2}r^{2}-A^{4}\right)\\ & +\left(\delta_{ki}r_{,j}r_{,l}+\delta_{kj}r_{,i}r_{,l}+\delta_{li}r_{j}r_{,k}+\delta_{lj}r_{,i}r_{,k}\right)(35r^{4}\\ & -14A^{2}r^{2}+3A^{4})\}]\;r>A, \end{array}$$

(76) $$\begin{array}{ll} \varepsilon_{ijkl}^{\left[3\right]A}\left(p,q\right)= & \frac{1}{7560\left(1-v\right)}[9v\delta_{ij}\{\delta_{kl}\left(-r^{4}+14A^{2}r^{2}+35A^{4}\right)\\ & +4r_{,k}r_{,l}r^{2}\left(-r^{2}+7A^{2}\right)\}-\left(\delta_{ij}\delta_{kl}+\delta_{ik}\delta_{jl}+\delta_{il}\delta_{jk}\right)\\ & \times \left(-r^{4}+18A^{2}r^{2}+63A^{4}\right)-4(\delta_{kl}r_{,i}r_{,j}+\delta_{jl}r_{,i}r_{,k}+\delta_{jk}r_{,i}r_{,l}\\ & +\delta_{il}r_{,j}r_{,k}+\delta_{ik}r_{,j}r_{,l}+\delta_{ij}r_{,k}r_{,l}r^{2}\left(-r^{2}+9A^{2}\right)+8r_{,i}r_{j}r_{,k}r_{,l}r^{4}\\ & +9\left(1-v\right)\{\left(\delta_{ik}\delta_{jl}+\delta_{il}\delta_{jk}\right)\left(-r^{4}+14A^{2}r^{2}+35A^{4}\right)+2(\delta_{jl}r_{,i}r_{,k}\\ & +\delta_{jk}r_{,i}r_{,l}+\delta_{il}r_{,j}r_{,k}+\delta_{ik}r_{,j}r_{,l})r^{2}\left(-r^{2}+7A^{2}\right)\}]\;r\leq A. \end{array}$$

The first thermal load is $T_{S}$, the final thermal load is $T_{0}$, and the number of iterations is N. Then, the incremental load is $\left(T_{0}-T_{S}\right)/N$.

The following iterative relationship is used to solve the current thermo-elastoplastic problem:

(77) $$\sigma_{0}^{k+1}=\sigma_{0}^{k}+Hd\varepsilon_{e}^{P},\;$$

where $\sigma_{0}^{k},\;\sigma_{0}^{k+1},\;H$, and $d\varepsilon_{e}^{P}$ are yield stress at k, yield stress at k+1, strain hardening, and equivalent plastic strain increment, respectively. Based on the von Mises yield criterion, the stresses rate in Equation (72) yields the deviatoric stress tensor $S_{ij}$, and the equivalent stress $\sigma_{e}$ can be computed as

(78) $$\sigma_{e}=\sqrt{\frac{3}{2}S_{ij}S_{ij}}\;$$

where

(79) $$\sigma_{e}-\sigma_{0}=0.$$

The following Prandtl–Reuss equation is employed to calculate the plastic strain increment $d\varepsilon_{ij}^{p}$ as

(80) $$d\varepsilon_{ij}^{p}=S_{ij}\;d\lambda ,\;$$

where dλ is a proportionality factor.

The plastic strain increment $d\varepsilon_{ij}^{p}$ is calculated using Equation (80).

Equations (36) and (37) are used to interpolate the initial strain rate.

The displacement and traction rates are calculated by Equation (43).

Equation (80) is used to calculate the strain rate.

Equation (77) is used to calculate the initial strain rate until convergence.

## 4. Numerical Results and Discussion

The proposed BEM method is general because it can be used to deal with a wide range of fractional thermo-elastoplastic problems affecting anisotropic fiber-reinforced polymer composite materials. Additionally, it is simple because only the surface of the domain needs to be discretized.

In our study computations, we employed a fiber-reinforced polymer composite with the following properties:

Young’s modulus E=210 GPa, Poisson’s ratio v=0.3, thermal expansion α=0.000011, yield stress $\sigma_{0}=250\;\mathrm{Mpa}$, and strain hardening H=0.05 E.

We considered the reinforcing parameters $\bar{\alpha }$, $\bar{\beta }$*,* and $\left({\bar{\mu }}_{L}-{\bar{\mu }}_{T}\right)$.

The pure anisotropic fiber-reinforced behavior satisfies

(81) $$c_{ijkl}u_{k,l}=⟦\bar{\lambda }\varepsilon_{kk}\delta_{ij}+2{\bar{\mu }}_{T}\varepsilon_{ij}+\bar{\alpha }\left({\bar{a}}_{k}{\bar{a}}_{m}\varepsilon_{km}\delta_{ij}+{\bar{a}}_{i}{\bar{a}}_{j}\varepsilon_{kk}\right)+2\left({\bar{\mu }}_{L}-\;{\bar{\mu }}_{T}\right)\left({\bar{a}}_{i}{\bar{a}}_{k}\varepsilon_{kj}+{\bar{a}}_{j}{\bar{a}}_{k}\varepsilon_{ki}\right)+\bar{\beta }{\bar{a}}_{k}{\bar{a}}_{m}\varepsilon_{km}a_{i}a_{j}⟧,\;\left(i,\;j,\;k,\;m=1,\;2,\;3\right),$$

where $a\equiv \left(a_{1},{\;a}_{2},{\;a}_{3}\right),{\;a}_{1}^{2}+a_{2}^{2}+a_{3}^{2}$

Additionally, the isotropic behavior satisfies $\bar{\alpha }=\bar{\beta }=\left({\bar{\mu }}_{L}-{\bar{\mu }}_{T}\right)=0$.

**Figure 1:**
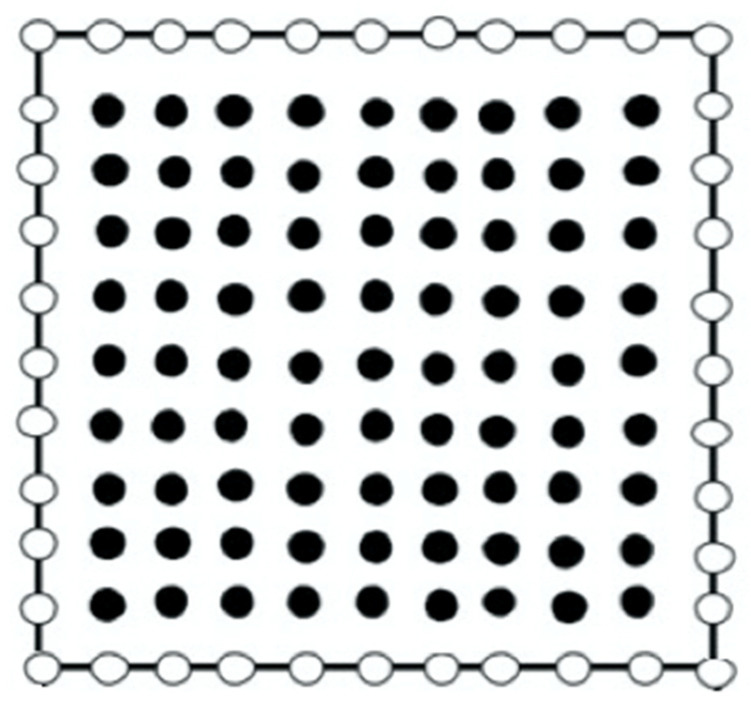
BEM modeling of the present problem.

As illustrated in Figure 1, the domain of the considered 3D problem includes 40 boundary nodes and 81 internal nodes. Additionally, we assumed that the wave direction is parallel to the $x_{1}$-axis.

**Figure 2:**
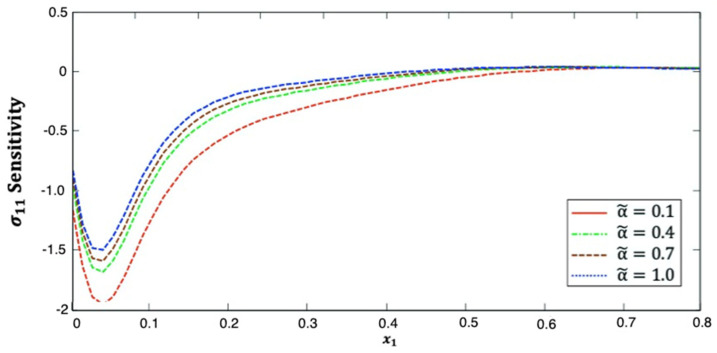
Distribution of the $\sigma_{11}$ sensitivity along $x_{1}–$axis in anisotropic FRP composites for various fractional-order values.

Figure 2 shows the distribution of the stress $\sigma_{11}$ sensitivity along the $x_{1}–$axis in anisotropic fiber-reinforced polymer composites for various fractional-order values. It is shown from this figure that the stress $\sigma_{11}$ sensitivity decreases and then increases along the $x_{1}–$axis. Additionally, it increases as the fractional-order parameter increases. This figure demonstrates that the fractional-order parameter has a significant effect on stress $\sigma_{11}$ sensitivity in anisotropic FRP composites. The stress $\sigma_{11}$ sensitivity curves at the upper ($\tilde{\alpha }=1.0$) and lower $(\tilde{\alpha }=0.1$) values of the fractional parameter diverge from each other, and they are close to each other at the interface values ($\tilde{\alpha }=0.4\;\mathrm{and}\;\tilde{\alpha }=0.7$).

**Figure 3:**
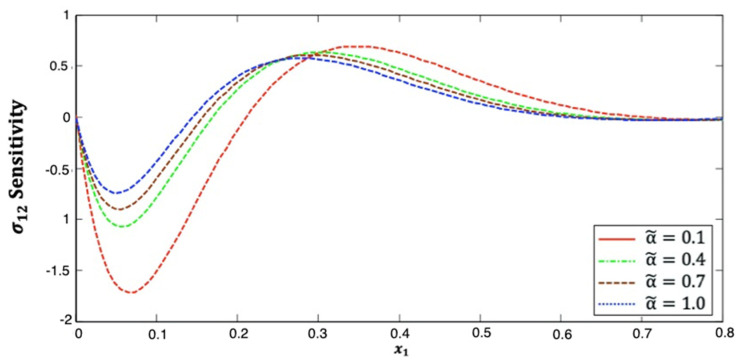
Distribution of the $\sigma_{12}$ sensitivity along $x_{1}–$axis in anisotropic FRP composites for various fractional-order values.

Figure 3 shows the distribution of the stress $\sigma_{12}$ sensitivity along the $x_{1}–$axis in anisotropic fiber-reinforced polymer composites for various fractional-order values. It is shown from this figure that the stress $\sigma_{12}$ sensitivity decreases and then increases and then decreases again the along $x_{1}–$axis. Additionally, it increases as the fractional-order parameter increases. This figure demonstrates that the fractional-order parameter has a significant effect on the stress $\sigma_{12}$ sensitivity in anisotropic FRP composites. The stress $\sigma_{12}$ sensitivity curves at the upper ($\tilde{\alpha }=1.0$) and lower $(\tilde{\alpha }=0.1$) values of the fractional parameter diverge from each other, and they are close to each other at the interface values ($\tilde{\alpha }=0.4\;\mathrm{and}\;\tilde{\alpha }=0.7$).

**Figure 4:**
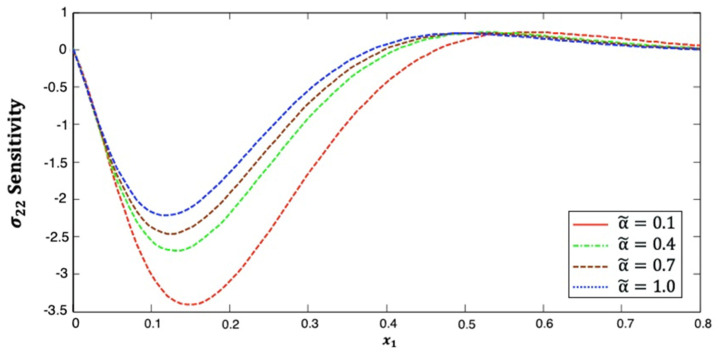
Distribution of the $\sigma_{22}$ sensitivity along $x_{1}–$axis in anisotropic FRP composites for various fractional-order values.

Figure 4 illustrates the distribution of the stress $\sigma_{22}$ sensitivity along the $x_{1}–$axis in anisotropic fiber-reinforced polymer composites for various fractional-order values. It is shown from this figure that the stress $\sigma_{22}$ sensitivity decreases and then increases along the $x_{1}–$axis. Additionally, it increases as the fractional-order parameter increases. This figure demonstrates that the fractional-order parameter has a significant effect on the stress $\sigma_{22}$ sensitivity in anisotropic FRP composites. The stress $\sigma_{22}$ sensitivity curves at the upper ($\tilde{\alpha }=1.0$) and lower $(\tilde{\alpha }=0.1$) values of the fractional parameter diverge from each other, and they are close to each other at the interface values ($\tilde{\alpha }=0.4\;\mathrm{and}\;\tilde{\alpha }=0.7$).

**Figure 5:**
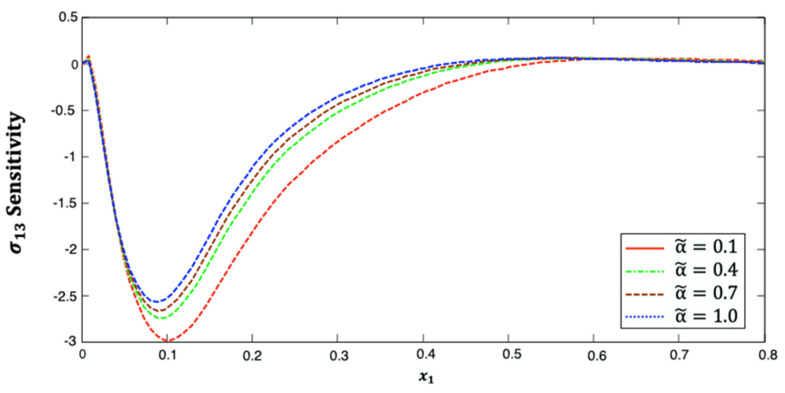
Distribution of the $\sigma_{13}$ sensitivity along $x_{1}–$axis in anisotropic FRP composites for various fractional-order values.

Figure 5 illustrates the distribution of the stress $\sigma_{13}$ sensitivity along the $x_{1}–$axis in anisotropic fiber-reinforced polymer composites for various fractional-order values. It is shown from this figure that the stress $\sigma_{13}$ sensitivity decreases and then increases along the $x_{1}–$axis. Additionally, it increases as the fractional-order parameter increases. This figure demonstrates that the fractional-order parameter has a significant effect on stress $\sigma_{13}$ sensitivity in anisotropic FRP composites. The stress $\sigma_{13}$ sensitivity curves at the upper ($\tilde{\alpha }=1.0$) and lower $(\tilde{\alpha }=0.1$) values of the fractional parameter diverge from each other, and they are close to each other at the interface values ($\tilde{\alpha }=0.4\;\mathrm{and}\;\tilde{\alpha }=0.7$).

**Figure 6:**
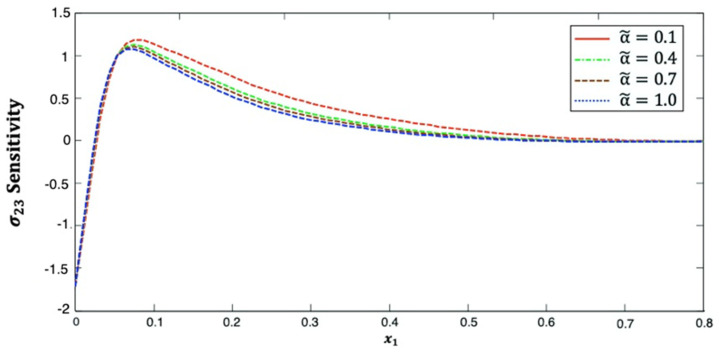
Distribution of the $\sigma_{23}$ sensitivity along $x_{1}–$axis in anisotropic FRP composites for various fractional-order values.

Figure 6 illustrates the distribution of the stress $\sigma_{23}$ sensitivity along the $x_{1}–$axis in anisotropic fiber-reinforced polymer composites for various fractional-order values. It can be seen from this figure that the stress $\sigma_{23}$ sensitivity increases and then decreases as $x_{1}$ increases for different fractional-order parameters. This figure demonstrates that the fractional-order parameter has a significant effect on the stress $\sigma_{23}$ sensitivity in anisotropic FRP composites. The stress $\sigma_{23}$ sensitivity curves at the upper ($\tilde{\alpha }=1.0$) and lower $(\tilde{\alpha }=0.1$) values of the fractional parameter diverge from each other, and they are close to each other at the interface values ($\tilde{\alpha }=0.4\;\mathrm{and}\;\tilde{\alpha }=0.7$).

**Figure 7:**
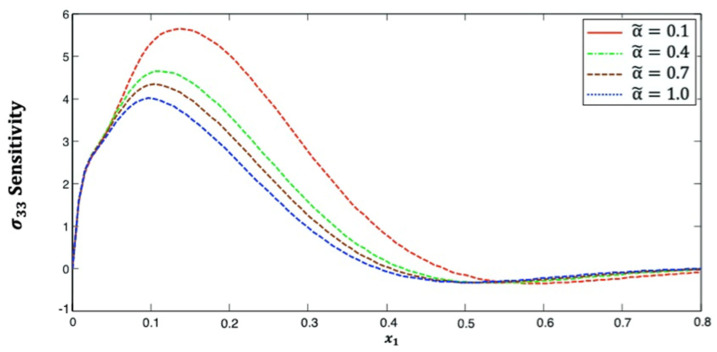
Distribution of the $\sigma_{33}$ sensitivity along $x_{1}–$axis in anisotropic FRP composites for various fractional-order values.

Figure 7 displays the distribution of stress $\sigma_{33}$ sensitivity along the $x_{1}–$axis in anisotropic fiber-reinforced polymer composites for various fractional-order values. The stress component $\sigma_{33}$ increases and then decreases as $x_{1}$ increases. This figure demonstrates that the fractional-order parameter has a significant effect on the stress $\sigma_{33}$ sensitivity in anisotropic FRP composites. The stress $\sigma_{33}$ sensitivity curves at the upper ($\tilde{\alpha }=1.0$) and lower $(\tilde{\alpha }=0.1$) values of the fractional parameter diverge from each other, and they are close to each other at the interface values ($\tilde{\alpha }=0.4\;\mathrm{and}\;\tilde{\alpha }=0.7$).

**Figure 8:**
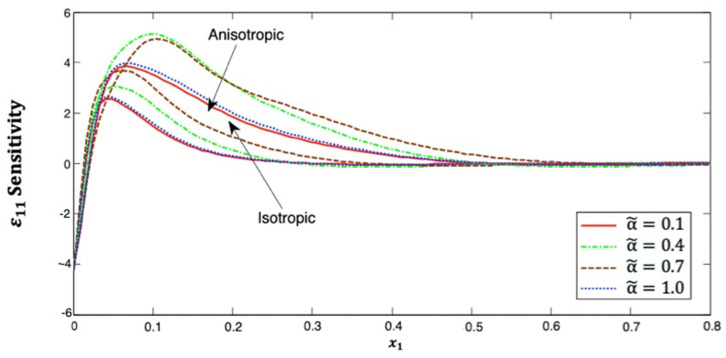
Distribution of the $\varepsilon_{11}$ sensitivity along $x_{1}–$axis in isotropic and anisotropic FRP composites for various fractional-order values.

Figure 8 explains the distribution of the strain $\varepsilon_{11}$ sensitivity along the $x_{1}–$axis, which, in isotropic and anisotropic cases, begins with a negative value. It can be seen from this figure that the distribution of the strain $\varepsilon_{11}$ sensitivity initially increases and then decreases along the $x_{1}–$axis. Additionally, it has $\tilde{\alpha }=0.7>\tilde{\alpha }=0.4>\tilde{\alpha }=1.0>\tilde{\alpha }=0.1$ in anisotropic cases but $\tilde{\alpha }=0.4>\tilde{\alpha }=0.7>\tilde{\alpha }=1.0>\tilde{\alpha }=0.1$ for isotropic cases. This figure demonstrates that the fractional-order parameter has a significant effect on the strain $\varepsilon_{11}$ sensitivity in both isotropic and anisotropic cases. The strain $\varepsilon_{11}$ sensitivity curves at the upper ($\tilde{\alpha }=1.0$) and lower $(\tilde{\alpha }=0.1$) values of the fractional parameter are also close to each other, and we notice that they are closer in the isotropic case than in the anisotropic case. It is demonstrated that the strain $\varepsilon_{11}$ sensitivity curves at the interface values diverge from each other, as they are further away in the isotropic case than in the anisotropic case.

**Figure 9:**
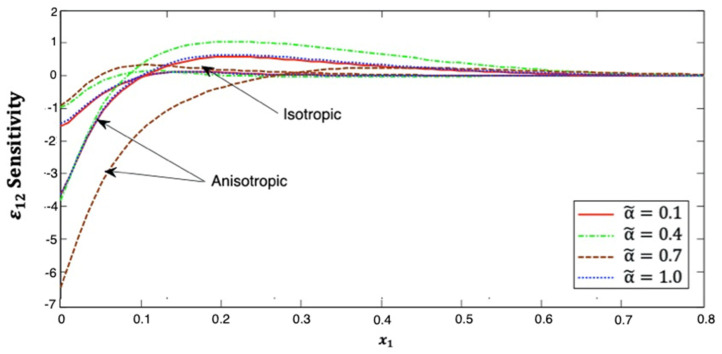
Distribution of the $\varepsilon_{12}$ sensitivity along $x_{1}–$axis in isotropic and anisotropic FRP composites for various fractional-order values.

Figure 9 illustrates the distribution of the strain $\varepsilon_{12}$ sensitivity along the $x_{1}–$axis in the context of isotropic and anisotropic fiber-reinforced polymer composites for various fractional-order values. It can be noticed from this figure that the strain $\varepsilon_{12}$ sensitivity increases as $x_{1}$ increases at small $x_{1}$ values. Additionally, it has $\tilde{\alpha }=0.4>\tilde{\alpha }=1.0>\tilde{\alpha }=0.1>\tilde{\alpha }=0.7$ in anisotropic cases, but it has $\tilde{\alpha }=0.7>\tilde{\alpha }=0.4>\tilde{\alpha }=1.0>\tilde{\alpha }=0.1$ in isotropic cases, which are close to the approximate values as $x_{1}$ tends to infinity. This figure demonstrates that the fractional-order parameter has an important effect on the strain $\varepsilon_{12}$ sensitivity in both isotropic and anisotropic cases. The strain $\varepsilon_{12}$ sensitivity curves at the upper ($\tilde{\alpha }=1.0$) and lower $(\tilde{\alpha }=0.1$) values of the fractional parameter are congruent in both cases. It is demonstrated that the strain $\varepsilon_{12}$ sensitivity curves at the interface values diverge from each other, as they are further away in the anisotropic case than in the isotropic case.

**Figure 10:**
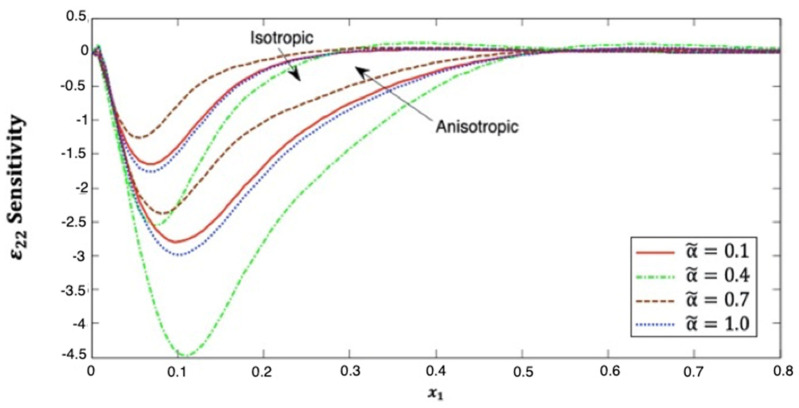
Distribution of the $\varepsilon_{22}$ sensitivity along $x_{1}–$axis in isotropic and anisotropic FRP composites for various fractional-order values.

Figure 10 explains the distribution of the strain $\varepsilon_{22}$ sensitivity along the $x_{1}–$axis, which starts near zero at $x_{1}=0$ in the context of both isotropic and anisotropic cases. It is noticed that distribution of the strain $\varepsilon_{22}$ sensitivity first decreases then increases as $x_{1}$ increases at small $x_{1}$ values. Additionally, it has $\tilde{\alpha }=0.7>\tilde{\alpha }=0.1>\tilde{\alpha }=1.0>\tilde{\alpha }=0.4$ in isotropic and anisotropic cases.

This figure demonstrates that the fractional-order parameter has a significant effect on the strain $\varepsilon_{22}$ sensitivity in both isotropic and anisotropic cases. The strain $\varepsilon_{22}$ sensitivity curves at the upper ($\tilde{\alpha }=1.0$) and lower $(\tilde{\alpha }=0.1$) values of the fractional parameter are also close to each other, and we notice that they are closer in the anisotropic case than in the isotropic case. It is demonstrated that the strain $\varepsilon_{22}$ sensitivity curves at the interface values diverge from each other, as they are further away in the anisotropic case than in the isotropic case.

**Figure 11:**
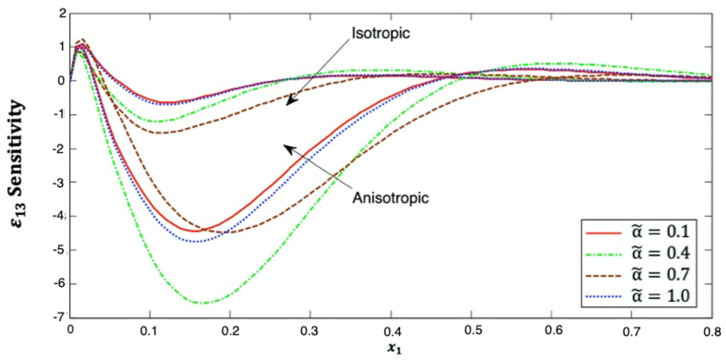
Distribution of the $\varepsilon_{13}$ sensitivity along $x_{1}–$axis in isotropic and anisotropic FRP composites for various fractional-order values.

Figure 11 depicts the distribution of the strain $\varepsilon_{13}$ sensitivity along the $x_{1}–$axis, which starts from zero at $x_{1}=0$ in the context of isotropic and anisotropic cases. It noticed that the strain $\varepsilon_{13}$ sensitivity is increases first and decreases and then increases again was $x_{1}$ increases. Additionally, it has $\tilde{\alpha }=0.1>\tilde{\alpha }=1.0>\tilde{\alpha }=0.4>\tilde{\alpha }=0.7$ for isotropic cases and $\tilde{\alpha }=0.7>\tilde{\alpha }=0.1>\tilde{\alpha }=1.0>\tilde{\alpha }=0.4$ for anisotropic cases. This figure demonstrates that the fractional-order parameter has a significant effect on the strain $\varepsilon_{13}$ sensitivity in both isotropic and anisotropic cases. The strain $\varepsilon_{13}$ sensitivity curves at the upper ($\tilde{\alpha }=1.0$) and lower $(\tilde{\alpha }=0.1$) values of the fractional parameter are also close to each other, and we notice that they are closer in the anisotropic case than in the isotropic case. It is demonstrated that the strain $\varepsilon_{13}$ sensitivity curves at the interface values diverge from each other, as they are further away in the anisotropic case than in the isotropic case.

**Figure 12:**
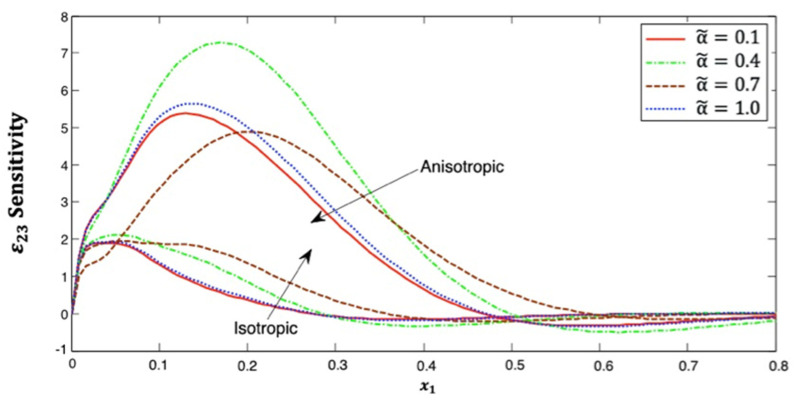
Distribution of the $\varepsilon_{23}$ sensitivity along $x_{1}–$axis in isotropic and anisotropic FRP composites for various fractional-order values.

Figure 12 explains the distribution of the strain $\varepsilon_{23}$ sensitivity along the $x_{1}–$axis, which starts near zero at $x_{1}=0$ in the context of isotropic and anisotropic fiber-reinforced polymer composites for various fractional-order values. It can be seen from this figure that the distribution of strain $\varepsilon_{23}$ sensitivity initially increases and then decreasing along the $\;x_{1}–$axis. Additionally, it has $\tilde{\alpha }=0.7>\tilde{\alpha }=0.4>\tilde{\alpha }=1.0>\tilde{\alpha }=0.1$ in isotropic cases but $\tilde{\alpha }=0.4>\tilde{\alpha }=1.0>\tilde{\alpha }=0.1>\tilde{\alpha }=0.7$ in anisotropic cases. This figure demonstrates that the fractional-order parameter has a significant effect on the strain $\varepsilon_{23}$ sensitivity in both isotropic and anisotropic cases. The strain $\varepsilon_{23}$ sensitivity curves at the upper ($\tilde{\alpha }=1.0$) and lower $(\tilde{\alpha }=0.1$) values of the fractional parameter are also close to each other, and we notice that they are closer in the anisotropic case than in the isotropic case. It is demonstrated that the strain $\varepsilon_{23}$ sensitivity curves at the interface values diverge from each other, as they are further away in the anisotropic case than in the isotropic case.

**Figure 13:**
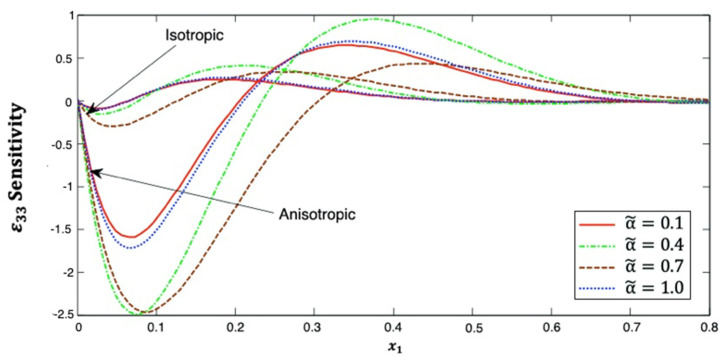
Distribution of the $\varepsilon_{33}$ sensitivity along $x_{1}–$axis in isotropic and anisotropic FRP composites for various fractional-order values.

Figure 13 depicts the distribution of strain $\varepsilon_{33}$, which starts from zero at $x_{1}=0$ in the context of isotropic and anisotropic cases. It noticed that the distribution decreases and then increases as $x_{1}$ increases at small $x_{1}$ values. Additionally, it has $\tilde{\alpha }=0.1>\tilde{\alpha }=1.0>\tilde{\alpha }=0.4>\tilde{\alpha }=0.7$ in both isotropic and anisotropic cases. This figure demonstrates that the fractional-order parameter has a significant effect on the strain $\varepsilon_{23}$ sensitivity in both isotropic and anisotropic cases. The strain $\varepsilon_{23}$ sensitivity curves at the upper ($\tilde{\alpha }=1.0$) and lower $(\tilde{\alpha }=0.1$) values of the fractional parameter are also close to each other, and we notice that they are closer in the anisotropic case than in the isotropic case. It is demonstrated that the strain $\varepsilon_{23}$ sensitivity curves at the interface values diverge from each other, as they are further away in the anisotropic case than in the isotropic case.

**Figure 14:**
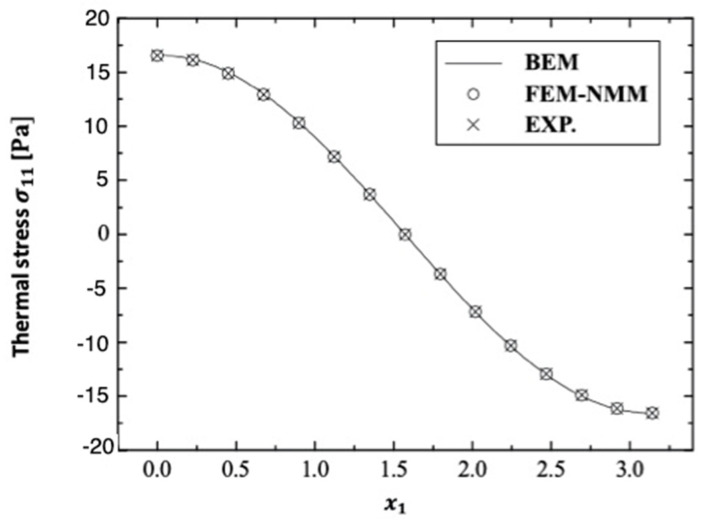
Distribution of the thermal stress wave $\sigma_{11}$ along $x_{1}–$axis in the special case of anisotropic FRP composites for BEM, FEM-NMM, and Exp.

**Figure 15:**
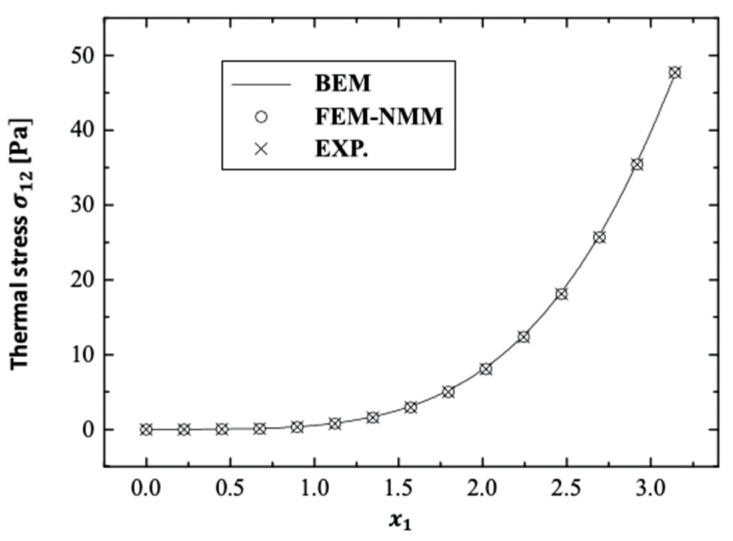
Distribution of the thermal stress wave $\sigma_{12}$ along $x_{1}–$axis in the special case of anisotropic FRP composites for BEM, FEM-NMM, and Exp.

**Figure 16:**
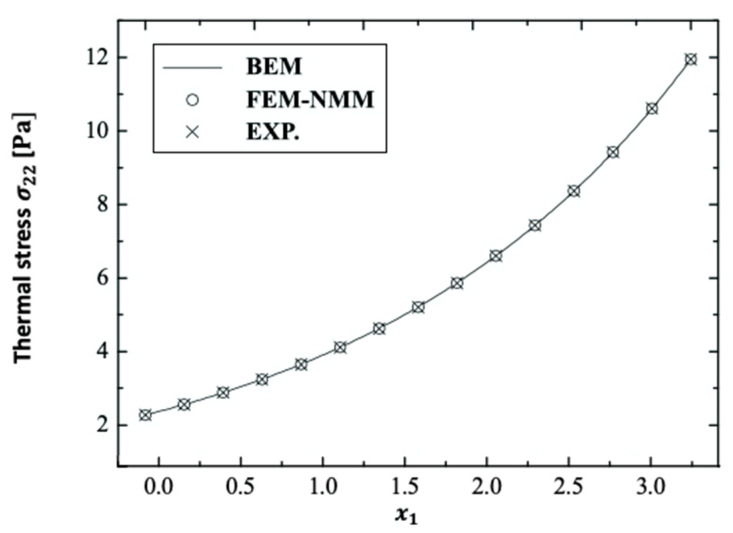
Distribution of the thermal stress wave $\sigma_{22}$ along $x_{1}–$axis in the special case of anisotropic FRP composites for BEM, FEM-NMM, and Exp.

There are no published results that demonstrate the validity and accuracy of the current BEM method strategy. On the other hand, some studies can be thought of as special cases in the context of this current general study. The special case distributions $\sigma_{11}$, $\sigma_{12}$, and $\sigma_{22}$ for the considered BEM combined the finite element method/normal mode method (FEM–NMM) of An et al. [23] and the experimental technique (Exp.) of Solodov et al. [24] and are shown in Figure 14, Figure 15 and Figure 16 for fractional-order ($\tilde{\alpha }=0.4$) anisotropic fiber-reinforced polymer composites. These results show that the BEM findings are in excellent agreement with those of FEM–NMM [23] and Exp. [24]. As a result, the validity of the proposed technique was confirmed.

## 5. Conclusions

The following findings can be drawn from the present paper:

1. Advanced BEM was applied to solve fractional-order thermo-elastoplastic ultrasonic wave propagation problems affecting anisotropic fiber-reinforced polymer composite materials
2. The Laplace transform was used to eliminate the time variable from the governing equations.
3. The unit step test function was used to derive the local boundary integral equations.
4. The MLS scheme was developed to treat the domain integrals and approximate physical quantities.
5. The numerical data demonstrate the current MLS approach’s accuracy, feasibility, effectiveness, and convergence.
6. The inverse Laplace transformation method was then used to find the transient temperature solutions.
7. The current technique’s main advantage is its generality and simplicity.
8. The initial stress and strain distributions are interpolated using boundary integral equations.
9. Numerical results show that the fractional-order parameter and anisotropy have significant effects on the thermoelastic behavior of fiber-reinforced polymer composites.
10. The numerical results show that the proposed strategy outperforms previous experimental and numerical methods.
11. The findings presented in this paper may be of interest to researchers in material science, mathematical physics, and geothermal engineering as well as those working on the development of anisotropic fiber-reinforced polymer composite materials.

## Data Availability

All data generated or analyzed during this study are included in the published article.

## Nomenclature

$\varepsilon_{ij}$	Strain
$\bar{\lambda }$ & ${\bar{\mu }}_{T}$	Elastic parameters
$\rho \left(x\right)$	Mass density
$\theta \left(x,t\right)$	Temperature field
$\sigma_{ij}$	Mechanical stress tensor
$\tau_{1}$	Laser pulse time characteristic
$\phi_{j}^{M}$	MLS shape functions
α	Thermal expansion
$\tilde{\alpha }$	Fractional-order parameter
$c\left(x\right)$	Specific heat
$c_{ijkl}$	Constant elastic moduli
$E_{i}$	Young’s moduli
G	Shear moduli
$J\left(\tau \right)$	Non-Gaussian temporal profile
$J_{0}$	Total energy intensity
$k_{ij}$	Thermal conductivity tensor
$n_{i}$	Unit normal vector
$Q\left(x,t\right)$	Heat source intensity
R	Irradiated surface absorptivity
v	Poisson’s ratios
